# Supplementary material for: Phenotypic Response to Light Versus Shade Associated with DNA Methylation Changes in Snapdragon Plants (Antirrhinum majus)
Source: Genes (Basel). 2021 Feb 4;12(2):227. doi: 10.3390/genes12020227 (PMC7914928; doi:10.3390/genes12020227)
Supplement: Supplementary file 1 [file genes-12-00227-s001.pdf]

Supplementary for:

Phenotypic response to light versus shade associated with DNA methylation changes in Snapdragon plants (*Antirrhinum majus*)

By Pierick Mouginot, Nelia Luviano Aparicio, Delphine Gourcilleau, Mathieu Latutrie, Sara Marin, Jean-Louis Hemptinne, Christoph Grunau, Benoit Pujol

Photo

Experimental garden at ENSFEA at Castanet-Tolosan (France).

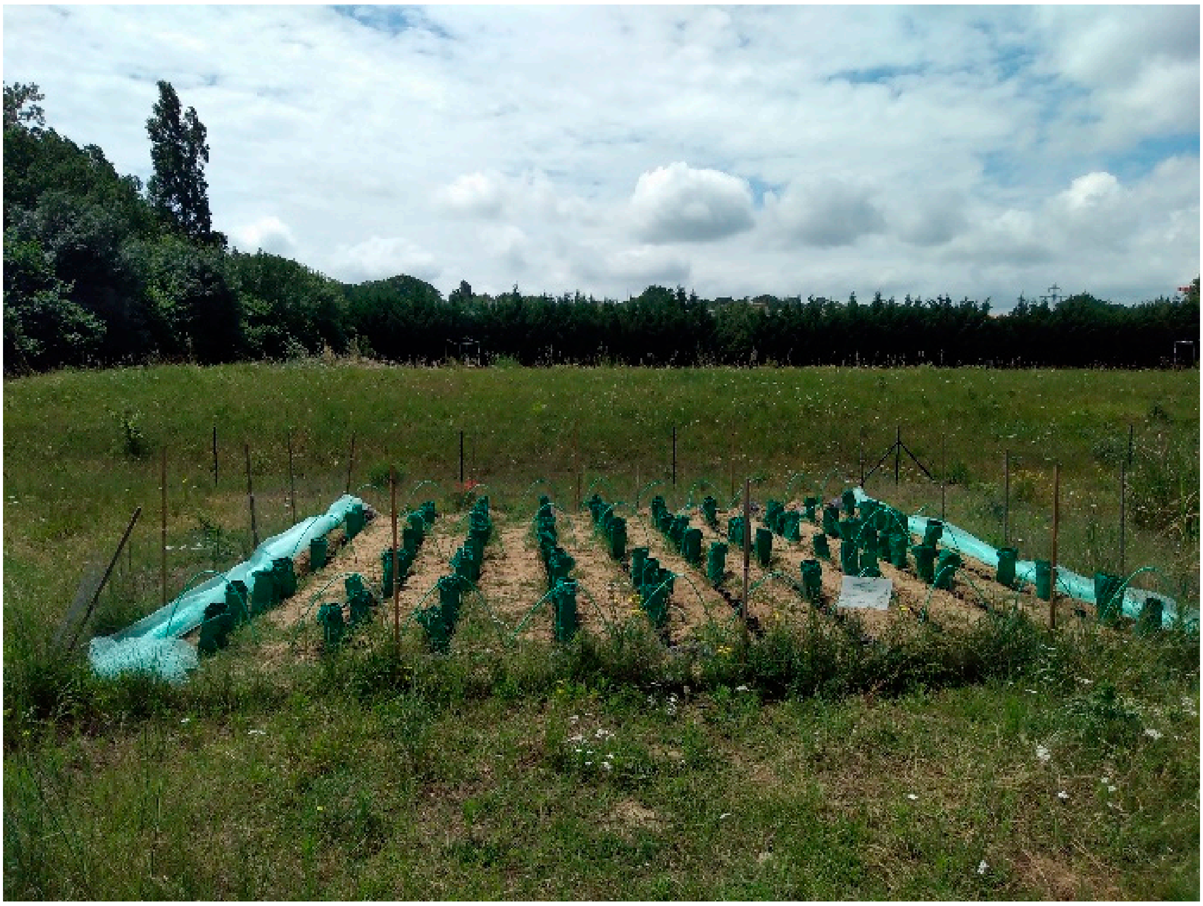

epiGBS protocol

The DNA content of 96 DNA isolated samples (2 tissues * 12 plants * 4 inbred lines) were quantified by using a Qubit fluorometer with the dsDNA HS Assay Kit (Invitrogen). 235 ng of DNA per sample were used for epiGBS benchmarking. DNA samples were digested overnight at 37°C with two combinations of restriction enzymes: Csp6I*NsiI and AseI*NsiI (Thermo Scientific, Waltham, Massachussetts, US for Csp6I and NEB, Ipswich Massachussetts, US for others). After digestion, ligation of distinctive barcoded

adapters per sample was done by using T4 DNA ligase (NEB, M202M/L), an incubation during overnight at 22°C. Following adapter ligation, all samples were pooled in one tube and then split in 8 tubes to proceed with concentration and cleaning with NucleoSpin® PCR Cleanup Kit (Macherey-Nagel). Then, DNA concentration was quantified by Qubit Assay. 0.8x SPRI size-selection was done with AMPure XP SPRI beads (Beckman Coulter, A63880). The nick repair step was done by a PCR reaction with a 5-methylcytosine dNTP Mix (Zymo Research, D1030). DNA was bisulfite converted with the EZ DNA methylation-Lightning Zymo Kit™ (Zymo Research) following manufacturer's instructions. Bisulfite-converted DNA was then amplified using KAPA HiFi HotStart Uracil+ ReadyMix (Kapa Biosystems) and setting the temperature cycling at 95°C for 3 min followed by 14 cycles of 98°C for 10s, 65°C for 15 s, 72°C for 15 s and a final extension step at 72°C for 5 min. Library was cleaned and concentrated with NucleoSpin® PCR Cleanup Kit and a final SPRI cleanup was done to eliminate any adapter dimer. Library concentration was determined by KAPA Library Quantification Kit Ilumina® (Kapa Biosystems). Fragment distribution was checked in a Hi-Sense Bioanalyzer 2100 chip (Agilet). Paired-end 150 bp sequencing was conducted on an Illumina HiSeq 2500 system.

Power analyses:

We estimated the power of the Mann-Whitney tests comparing the shade and light treatment groups with the R package "wmwpow" (Mollan et al. 2020). We estimated the power of the Spearman correlations between phenotypic traits and methylation PCA coordinates with the R package "pwr" (Champely 2020). We used the Spearman correlation coefficient (r_S) as if it were Pearson coefficient (r_P) (Myers & Sirois 2006).

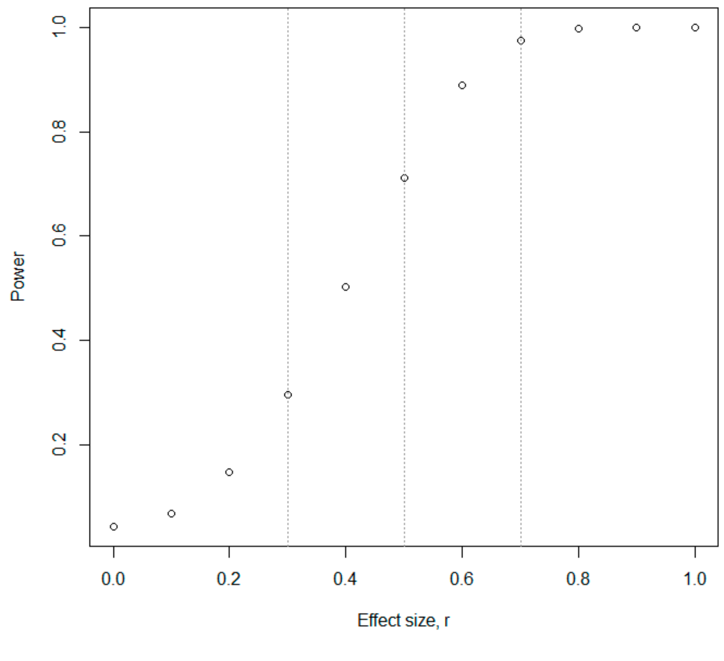

Figure S1: Power as a function of the effect size (*r*) in the case of our Mann-whitney tests for light treatment effect (n = 6 in each treatment group). Dotted lines represent r = 0.3, r = 0.5 and r = 0.7.

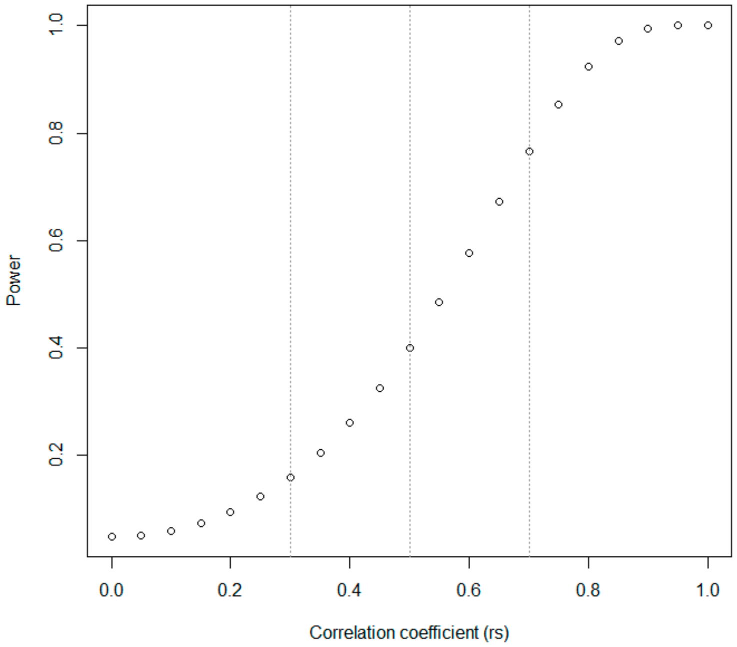

Figure S2: Power as a function of the Spearman correaltion coefficient (r_s) in the case of our correlations between phenotpypic trait and Methylation PCA coordinates (n = 12 in each treatment group). Dotted lines represent r_s = 0.3, r_s = 0.5 and r_s = 0.7.

Table S1: Phenotypic measurements (median [IQR]) in the shade and light treatments for each line, effect sizes of the treatment on phenotypic traits and their 95% confidence interval.

trait	line	shade [IQR]	light [IQR]	effect size (r)	95% CI
Ramification	165E	0 [0]	1.5 [1.75]	0.673	0.276; 0.927
	Ji75	1 [0.75]	1.5 [1]	0.626	0.303; 0.837
	Ji98	0 [0.75]	1.5 [1]	0.733	0.485; 0.909
	Si50	0 [0]	1 [0.75]	0.667	0.361; 0.958
Mean Internode length	165E	2.5 [0.25]	1.847 [0.785]	-0.419	-0.843; 0.214
	Ji75	2.604 [0.385]	1.604 [0.281]	-0.811	-0.846; -0.629
	Ji98	2.5 [0.469]	1.66 [0.203]	-0.65	-0.846; -0.14
	Si50	2.917 [0.542]	2.206 [0.105]	-0.84	-0.875; -0.647
Diameter	165E	0.95 [0.16]	1.615 [0.245]	0.834	0.638; 0.857
	Ji75	0.915 [0.195]	1.295 [0.305]	0.739	0.404; 0.846
	Ji98	0.89 [0.143]	1.395 [0.29]	0.834	0.635; 0.854
	Si50	0.89 [0.11]	1.78 [0.312]	0.831	0.632; 0.849
Total Leaves number	165E	10 [0.75]	33.5 [14.5]	0.745	0.407; 0.872
	Ji75	14 [5.25]	26 [3.75]	0.837	0.641; 0.866
	Ji98	10.5 [4.75]	33 [12.75]	0.834	0.638; 0.857
	Si50	8 [0]	26 [10]	0.889	0.73; 0.95
SLA	165E	75.57 [18.37]	22.478 [2.628]	-0.831	-0.849; -0.632
	Ji75	74.834 [8.512]	19.457 [4.39]	-0.831	-0.849; -0.632
	Ji98	87.364 [21.833]	17.845 [3.952]	-0.831	-0.849; -0.635
	Si50	81.545 [10.575]	23.221 [2.82]	-0.831	-0.849; -0.632

Height	165E	8.75 [2.25]	8 [2.25]	-0.257	-0.725; 0.358
	Ji75	7.5 [1]	6.25 [0.5]	-0.658	-0.866; -0.236
	Ji98	8 [1.375]	8 [1.125]	-0.259	-0.739; 0.381
	Si50	8.5 [0.375]	9.5 [2.125]	0.306	-0.358; 0.826

Table S2: Relative contribution of each dimension to the explanation of DNA methylation data variation presented for each PCA.

			Dimension											
tissue	protocol	line	1	2	3	4	5	6	7	8	9	10	11	12
Apex	CHH	165E	25.94	15.69	8.98	8.44	8.29	6.71	6.49	6.0	5.32	4.43	3.75	NA
		Ji75	23.61	13.42	13.39	10.58	8.72	7.91	6.56	4.82	4.43	3.68	2.89	NA
		Ji98	27.85	13.03	11.72	7.32	7.12	6.65	5.97	5.78	5.42	4.66	4.49	NA
		Si50	17.21	11.61	11.13	10.14	9.05	8.47	7.42	7.33	6.63	5.87	5.16	NA
	CHG	165E	14.25	11.61	10.3	9.78	9.71	9.09	8.75	7.44	7.01	6.39	5.68	NA
		Ji75	13.98	10.61	10.47	10.18	9.55	9.09	8.13	7.77	6.98	6.81	6.44	NA
		Ji98	14.78	13.10	11.61	9.73	8.87	8.42	7.65	7.42	6.69	6.20	5.52	NA
		Si50	22.23	10.74	10.47	9.43	8.55	7.84	6.95	6.86	6.09	5.99	4.88	NA
	CpG	165E	14.1	12.2	10.9	10.5	9.04	8.71	7.9	7.35	7.02	6.2	6.01	10^{-29}
		Ji75	19.2	15.1	9.9	9.23	8.53	7.69	7.28	6.47	6.02	5.56	4.99	10^{-29}
		Ji98	19.7	11.9	11.1	9.51	8.25	7.81	7.34	6.78	6.17	5.9	5.48	10^{-29}
		Si50	22	12.6	10.4	9.09	8.47	6.98	6.9	6.4	6	5.64	5.43	10^{-29}
Leaf	CHH	165E	31.7	12.05	11.03	9.73	8.6	7.37	6.15	4.57	3.83	3.32	1.66	NA
		Ji75	20.14	12.58	10.53	9.43	8.9	8.19	7.24	6.37	6.07	5.34	5.23	NA
		Ji98	16.87	12.52	11.48	8.63	8.31	8.21	7.74	7.12	6.91	6.43	5.78	NA
	CHG	165E	16.07	12.80	10.21	9.81	8.47	8.19	7.82	7.41	6.62	6.35	6.26	NA
		Ji75	17.21	12.35	10.74	9.13	8.4	8.05	7.2	7.06	6.97	6.71	6.19	NA
		Ji98	20.44	14.7	14.03	11.08	7.24	6.8	6.06	5.54	5.16	4.57	4.4	NA
	CpG	165E	13.2	11.8	10.7	9.27	8.91	8.21	8.08	7.96	7.57	7.36	6.87	10^{-29}
		Ji75	21.4	12.1	10.4	9.97	8.57	7.73	7.05	6.04	5.81	5.66	5.25	10^{-29}
		Ji98	12.6	11.9	11.1	9.7	8.9	8.48	8.31	8.23	8.05	6.68	5.97	10^{-29}

Table S3: Effect sizes of methylation differences between light treatments for each line, each PCA dimension and each methylation protocol (CHG, CHH, CpG) applied on apex tissue.

		Apex CHG		Apex CHH		Apex CpG	
Line	Dimension	Effect size	95% CI	Effect size	95% CI	Effect size	95% CI
165E	1	-0.277	-0.837; 0.401	0.603	-0.563; 0.603	0.231	-0.404; 0.753
	2	-0.0462	-0.598; 0.566	0.736	-0.45; 0.736	0.416	-0.193; 0.831
	3	-0.0462	-0.647; 0.548	0.497	-0.794; 0.497	0.647	0.197; 0.843
	4	0.323	-0.28; 0.794	0.843	0.281; 0.843	0.139	-0.459; 0.684
	5	-0.0924	-0.652; 0.52	0.595	-0.655; 0.595	-0.37	-0.837; 0.283
	6	-0.185	-0.707; 0.445	0.027	-0.837; 0.0272	0.162	-0.494; 0.748
	7	-0.37	-0.837; 0.28	0.693	-0.542; 0.693	-0.092	-0.699; 0.548
	8	0.0924	-0.592; 0.65	0.803	-0.294; 0.803	-0.046	-0.641; 0.563
	9	-0.416	-0.828; 0.167	0.445	-0.736; 0.445	-0.462	-0.837; 0.129

	10	-0.323	-0.811; 0.28	0.595	-0.629; 0.595	0.185	-0.404; 0.748
	11	-0.185	-0.701; 0.41	0.632	-0.644; 0.632	0.046	-0.546; 0.641
	12	-	-	-	-	0.37	-0.234; 0.831
Ji75	1	0.231	-0.407; 0.797	0.566	-0.725; 0.566	0.231	-0.401; 0.739
	2	-0.185	-0.8; 0.462	0.45	-0.687; 0.45	0	-0.629; 0.606
	3	0.462	-0.141; 0.84	0.751	-0.262; 0.751	-0.277	-0.803; 0.355
	4	0.0462	-0.52; 0.621	0.595	-0.603; 0.595	-0.046	-0.595; 0.548
	5	0.462	-0.118; 0.837	0.641	-0.563; 0.641	-0.462	-0.84; 0.119
	6	0.0462	-0.551; 0.606	0.217	-0.794; 0.217	0.277	-0.398; 0.837
	7	-0.139	-0.681; 0.497	0.566	-0.647; 0.566	-0.139	-0.8; 0.502
	8	0.231	-0.39; 0.736	0.644	-0.505; 0.644	0.323	-0.28; 0.797
	9	-0.0924	-0.65; 0.52	0.736	-0.468; 0.736	-0.323	-0.837; 0.358
	10	0.139	-0.563; 0.73	0.736	-0.398; 0.736	0.462	-0.099; 0.831
	11	0	-0.635; 0.595	0.165	-0.828; 0.165	-0.416	-0.831; 0.168
	12	-	-	-	-	-0.6	-0.843; -0.099
Ji98	1	0.139	-0.497; 0.794	0.794	-0.401; 0.794	-0.092	-0.635; 0.505
	2	0.139	-0.468; 0.655	0.843	0; 0.843	-0.462	-0.84; 0.129
	3	0.277	-0.355; 0.797	0.84	0.0494; 0.84	0	-0.612; 0.609
	4	-0.139	-0.661; 0.499	0.563	-0.629; 0.563	-0.37	-0.788; 0.248
	5	0.739	0.407; 0.846	0.84	-0.167; 0.84	0.323	-0.309; 0.826
	6	0	-0.595; 0.629	0.837	-0.398; 0.837	-0.231	-0.828; 0.497
	7	0.508	-0.027; 0.84	0.372	-0.797; 0.372	-0.37	-0.788; 0.214
	8	0.139	-0.456; 0.701	0.546	-0.641; 0.546	0.139	-0.456; 0.687
	9	-0.231	-0.733; 0.375	0.543	-0.603; 0.543	0.323	-0.264; 0.794
	10	0.0462	-0.551; 0.612	0.742	-0.505; 0.742	-0.092	-0.647; 0.543
	11	0.462	-0.15; 0.84	0.494	-0.687; 0.494	-0.139	-0.655; 0.497
	12	-	-	-	-	0.255	-0.358; 0.745
Si50	1	-0.139	-0.742; 0.499	0.828	-0.217; 0.828	0.092	-0.574; 0.707
	2	-0.416	-0.834; 0.168	0.826	-0.309; 0.826	0.277	-0.355; 0.797
	3	-0.0924	-0.647; 0.505	0.0805	-0.817; 0.0805	0.323	-0.251; 0.794
	4	-0.231	-0.782; 0.41	0.546	-0.638; 0.546	0.6	0.118; 0.84
	5	0.37	-0.282; 0.837	0.569	-0.73; 0.569	-0.277	-0.742; 0.355
	6	-0.323	-0.75; 0.262	0.401	-0.736; 0.401	0.185	-0.465; 0.794
	7	0.0462	-0.6; 0.727	0.638	-0.589; 0.638	0.139	-0.499; 0.782
	8	0.139	-0.497; 0.655	0.701	-0.407; 0.701	0.462	-0.166; 0.837
	9	0.231	-0.404; 0.794	0.603	-0.612; 0.603	-0.6	-0.843; 0
	10	0.323	-0.309; 0.828	-0.05	-0.837; -0.05	0.185	-0.47; 0.733
	11	0.323	-0.297; 0.826	0.621	-0.546; 0.621	-0.231	-0.794; 0.398
	12	-	-	-	-	0.462	-0.094; 0.831

Table S4: Effect sizes of methylation differences between light treatments for each line, each PCA dimension and each methylation protocol (CHG, CHH, CpG) applied on leaf tissue.

		Leaf CHG		Leaf CHH		Leaf CpG	
Line	Dimension	Effect size	95% CI	Effect size	95% CI	Effect size	95% CI
165E	1	-0.323	-0.831;0.264	0.393	-0.199; 0.794	-0.092	-0.649; 0.502
	2	-0.323	-0.745;0.262	0.116	-0.511; 0.736	0.416	-0.167; 0.825
	3	-0.185	-0.73;0.404	-0.439	-0.837; 0.166	0.231	-0.375; 0.805
	4	0.046	-0.566;0.649	0.255	-0.401; 0.82	-0.277	-0.745; 0.309
	5	0.37	-0.248;0.803	-0.3	-0.797; 0.323	-0.185	-0.748; 0.45
	6	0.092	-0.546;0.69	0.0232	-0.595; 0.641	-0.323	-0.794; 0.297
	7	0.508	-0.027;0.831	0.208	-0.392; 0.725	0.162	-0.45; 0.724
	8	-0.231	-0.745;0.387	-0.0696	-0.727; 0.598	-0.323	-0.794; 0.281
	9	-0.046	-0.655;0.563	-0.3	-0.837; 0.372	-0.416	-0.82; 0.166
	10	0.092	-0.499;0.794	-0.0696	-0.655; 0.56	-0.185	-0.687; 0.41
	11	0	-0.572;0.589	-0.277	-0.797; 0.312	0	-0.595; 0.595

	12	-	-	-	-	-0.58	-0.84; -0.0831
Ji75	1	-0.185	-0.713;0.444	0.0462	-0.563; 0.615	-0.37	-0.834; 0.245
	2	-0.139	-0.658;0.514	0.139	-0.456; 0.658	0.462	-0.095; 0.828
	3	0.0462	-0.583;0.595	-0.0924	-0.696; 0.499	-0.092	-0.647; 0.52
	4	0.0462	-0.554;0.641	0.323	-0.28; 0.8	-0.139	-0.655; 0.496
	5	0.647	0.2;0.843	-0.0924	-0.647; 0.517	0.185	-0.407; 0.733
	6	0.416	-0.165;0.831	-0.693	-0.846; -0.264	-0.416	-0.797; 0.15
	7	0.185	-0.456;0.736	0.231	-0.401; 0.748	0.046	-0.595; 0.603
	8	-0.185	-0.745;0.453	0.185	-0.45; 0.707	-0.185	-0.699; 0.407
	9	0.092	-0.511;0.644	-0.139	-0.751; 0.502	-0.231	-0.73; 0.404
	10	0	-0.595;0.595	0.323	-0.297; 0.794	0.0924	-0.499; 0.652
	11	0	-0.595;0.598	0	-0.595; 0.598	-0.185	-0.699; 0.407
	12	-	-	-	-	-0.139	-0.739; 0.502
Ji98	1	0.416	-0.167;0.834	-0.0924	-0.701; 0.531	0.65	0.2; 0.843
	2	-0.323	-0.802;0.279	0.277	-0.326; 0.736	-0.231	-0.745; 0.407
	3	0.462	-0.1;0.837	-0.554	-0.84; 0	-0.139	-0.684; 0.502
	4	0.139	-0.453;0.696	-0.185	-0.713; 0.421	0.231	-0.401; 0.753
	5	-0.37	-0.803;0.242	-0.139	-0.791; 0.499	0.0462	-0.589; 0.612
	6	-0.416	-0.837;0.213	-0.37	-0.837; 0.283	0.323	-0.297; 0.834
	7	0.092	-0.583;0.644	-0.323	-0.797; 0.284	-0.554	-0.84; 0
	8	0.139	-0.468;0.661	-0.277	-0.794; 0.375	0.185	-0.453; 0.756
	9	0.092	-0.502;0.647	0.0924	-0.508; 0.649	0.231	-0.398; 0.794
	10	0.185	-0.459;0.736	-0.0462	-0.609; 0.551	-0.231	-0.733; 0.355
	11	-0.046	-0.638;0.546	0	-0.595; 0.554	0.37	-0.213; 0.828
	12	-	-	-	-	-0.092	-0.647; 0.548

Table S5: Spearman correlation coefficients (r_s) between each phenotypic trait and methylation PCA coordinates presented for cases showing methylation differences in apical tissue.

traits	Methylation protocol	Line	Dimension	r_S	95% CI
Ramification	CHG	Ji98	5	0.62	0.073; 0.907
	CHH	165E	4	0.642	0.032; 0.957
	CHH	Ji98	3	0.644	-0.047; 0.952
	CHH	Si50	10	-0.643	-0.935; -0.087
	CpG	165E	3	0.561	0.05; 0.855
	CpG	Ji75	12	-0.685	-0.892; -0.241
	CpG	Si50	4	0.399	-0.209; 0.806
Mean Internode length	CHG	Ji98	5	-0.359	-0.818; 0.346
	CHH	165E	4	-0.29	-0.781; 0.31
	CHH	Ji98	3	0.139	-0.578; 0.739
	CHH	Si50	10	-0.136	-0.639; 0.544
	CpG	165E	3	-0.262	-0.847; 0.461
	CpG	Ji75	12	0.185	-0.419; 0.735
	CpG	Si50	4	-0.431	-0.841; 0.277
Diameter	CHG	Ji98	5	-0.785	-0.93; -0.376
	CHH	165E	4	-0.093	-0.692; 0.522
	CHH	Ji98	3	-0.144	-0.631; 0.462
	CHH	Si50	10	0.381	-0.222; 0.755
	CpG	165E	3	-0.221	-0.742; 0.484
	CpG	Ji75	12	0.571	-0.079; 0.891
	CpG	Si50	4	-0.698	-0.971; -0.119
Total Leaves number	CHG	Ji98	5	0.639	0.077; 0.852
	CHH	165E	4	0.392	-0.282; 0.874
	CHH	Ji98	3	0.515	-0.027; 0.85
	CHH	Si50	10	-0.412	-0.79; 0.179

	CpG	165E	3	0.75	0.364; 0.901
	CpG	Ji75	12	-0.46	-0.893; 0.468
	CpG	Si50	4	0.815	0.488; 0.913
SLA	CHG	Ji98	5	-0.748	-0.978; -0.193
	CHH	165E	4	-0.608	-0.864; -0.076
	CHH	Ji98	3	-0.427	-0.852; 0.174
	CHH	Si50	10	0.336	-0.293; 0.762
	CpG	165E	3	-0.531	-0.842; 0.071
	CpG	Ji75	12	0.524	0.011; 0.846
	CpG	Si50	4	-0.734	-0.907; -0.331
Height	CHG	Ji98	5	0.579	0.044; 0.85
	CHH	165E	4	0.57	-0.135; 0.913
	CHH	Ji98	3	0.754	0.285; 0.941
	CHH	Si50	10	-0.623	-0.823; -0.114
	CpG	165E	3	0.817	0.447; 0.955
	CpG	Ji75	12	-0.489	-0.878; 0.14
	CpG	Si50	4	0.717	0.238; 0.899

Table S6: Spearman correlation coefficients (r_s) between each phenotypic trait and methylation PCA coordinates presented for cases showing methylation differences in leaf tissue.

traits	Methylation protocol	Line	Dimension	r_S	95% CI
Ramification	CHG	Ji75	Dim.5	0.867	0.541; 0.978
	CHH	Ji75	Dim.6	-0.336	-0.805; 0.284
	CpG	165E	Dim.12	-0.657	-0.888; -0.143
	CpG	Ji98	Dim.1	0.686	0.17; 0.951
Mean Internode length	CHG	Ji75	Dim.5	-0.281	-0.799; 0.333
	CHH	Ji75	Dim.6	0.911	0.655; 0.978
	CpG	165E	Dim.12	-0.138	-0.735; 0.499
	CpG	Ji98	Dim.1	0	-0.662; 0.71
Diameter	CHG	Ji75	Dim.5	-0.581	-0.954; 0
	CHH	Ji75	Dim.6	0.574	0.043; 0.874
	CpG	165E	Dim.12	-0.198	-0.779; 0.439
	CpG	Ji98	Dim.1	-0.305	-0.814; 0.312
Total Leaves number	CHG	Ji75	Dim.5	0.217	-0.333; 0.724
	CHH	Ji75	Dim.6	-0.359	-0.793; 0.219
	CpG	165E	Dim.12	-0.393	-0.845; 0.237
	CpG	Ji98	Dim.1	0.338	-0.219; 0.749
SLA	CHG	Ji75	Dim.5	-0.601	-0.936; -0.029
	CHH	Ji75	Dim.6	0.545	-0.079; 0.74
	CpG	165E	Dim.12	0.287	-0.394; 0.793
	CpG	Ji98	Dim.1	-0.574	-0.901; 0.039
Height	CHG	Ji75	Dim.5	0.479	-0.093; 0.837
	CHH	Ji75	Dim.6	-0.634	-0.937; -0.047
	CpG	165E	Dim.12	-0.495	-0.93; 0.103
	CpG	Ji98	Dim.1	0.452	-0.151; 0.752

R SCRIPT: PCA_analysis CG_context.R

```
##Script for methylKit analysis##

library(methylKit)

#File list with the files to be analyzed together

file.list165E_L<- list("MethylKit_54_light.tabular", "MethylKit_40_light.tabular", "MethylKit_2_light.tabular",
                    "MethylKit_26_light.tabular", "MethylKit_126_light.tabular", "MethylKit_8_light.tabular",
                    "MethylKit_62_shade.tabular", "MethylKit_68_shade.tabular", "MethylKit_78_shade.tabular",
                    "MethylKit_110_shade.tabular", "MethylKit_116_shade.tabular", "MethylKit_128_shade.tabular")

file.list165E_A<- list("MethylKit_1_light.tabular", "MethylKit_7_light.tabular", "MethylKit_25_light.tabular",
                    "MethylKit_39_light.tabular", "MethylKit_53_light.tabular", "MethylKit_125_light.tabular",
                    "MethylKit_61_shade.tabular", "MethylKit_67_shade.tabular", "MethylKit_77_shade.tabular",
                    "MethylKit_109_shade.tabular", "MethylKit_115_shade.tabular", "MethylKit_127_shade.tabular")

file.listJi75_L<- list("MethylKit_34_light.tabular", "MethylKit_64_light.tabular", "MethylKit_76_light.tabular",
                    "MethylKit_84_light.tabular", "MethylKit_112_light.tabular", "MethylKit_136_light.tabular",
                    "MethylKit_60_shade.tabular", "MethylKit_80_shade.tabular", "MethylKit_86_shade.tabular",
                    "MethylKit_102_shade.tabular", "MethylKit_120_shade .tabular", "MethylKit_122_shade.tabular")

file.listJi75_A<- list("MethylKit_33_light.tabular", "MethylKit_63_light.tabular", "MethylKit_75_light.tabular",
```

```
                    "MethylKit_83_light.tabular", "MethylKit_111_light.tabular",
"MethylKit_135_light.tabular",

                    "MethylKit_59_shade.tabular", "MethylKit_79_shade.tabular",
"MethylKit_85_shade.tabular",

                    "MethylKit_101_shade.tabular", "MethylKit_119_shade.tabular",
"MethylKit_121_shade.tabular")


file.listJi98_L<- list("MethylKit_38_light.tabular", "MethylKit_56_light.tabular",
"MethylKit_66_light.tabular",

                    "MethylKit_74_light.tabular", "MethylKit_114_light.tabular",
"MethylKit_132_light.tabular",

                    "MethylKit_44_shade.tabular", "MethylKit_52_shade.tabular",
"MethylKit_92_shade.tabular",

                    "MethylKit_100_shade.tabular", "MethylKit_108_shade.tabular",
"MethylKit_124_shade.tabular")


file.listJi98_A<- list("MethylKit_13_light.tabular", "MethylKit_55_light.tabular",
"MethylKit_65_light.tabular",

                    "MethylKit_73_light.tabular", "MethylKit_113_light.tabular",
"MethylKit_131_light.tabular",

                    "MethylKit_43_shade.tabular", "MethylKit_51_shade.tabular",
"MethylKit_91_shade.tabular",

                    "MethylKit_99_shade.tabular", "MethylKit_107_shade.tabular",
"MethylKit_123_shade.tabular")


file.listSi50_A<- list("MethylKit_9_light.tabular", "MethylKit_15_light.tabular",
"MethylKit_27_light.tabular",

                    "MethylKit_35_light.tabular", "MethylKit_71_light.tabular", "MethylKit_89_light.tabular",

                    "MethylKit 3_shade.tabular", "MethylKit_41_shade.tabular",
"MethylKit_45_shade.tabular",

                    "MethylKit_47_shade.tabular", "MethylKit_49_shade.tabular",
"MethylKit_105_shade.tabular")


#Convert file list to methylKit objetcs


MyObj165E_L<- methRead(file.list165E_L,
```

```
        sample.id=list ("54", "40", "2", "26", "126", "8",
                "62", "68", "78_", "110", "116", "128"),
        assembly = "hg18", treatment = c(1, 1, 1, 1, 1, 1, 0, 0, 0, 0, 0, 0))

MyObj165E_A<- methRead(file.list165E_A,
          sample.id=list ("1", "7", "25", "39", "53", "125",
                  "61", "67", "77", "109", "115", "127"),
          assembly = "hg18", treatment = c(1, 1, 1, 1, 1, 1, 0, 0, 0, 0, 0, 0))

MyObjJi75_L<- methRead(file.listJi75_L,
          sample.id=list ("34", "64", "76", "84", "112", "136",
                  "60", "80", "86", "102", "120", "122"),
          assembly = "hg18", treatment = c(1, 1, 1, 1, 1, 1, 0, 0, 0, 0, 0, 0))

MyObjJi75_A<- methRead(file.listJi75_A,
          sample.id=list ("33", "63", "75", "83", "111", "135",
                  "59", "79", "85", "101", "119", "121"),
          assembly = "hg18", treatment = c(1, 1, 1, 1, 1, 1, 0, 0, 0, 0, 0, 0))

MyObjJi98_A<- methRead(file.listJi98_A,
          sample.id=list ("13", "55", "65", "73", "113", "131",
                  "43", "51", "91", "99", "107", "123"),
          assembly = "hg18", treatment = c(1, 1, 1, 1, 1, 1, 0, 0, 0, 0, 0, 0))

MyObjJi98_L<- methRead(file.listJi98_L,
          sample.id=list ("38", "56", "66", "74", "114", "132",
                  "44", "52", "92", "100", "108", "124"),
          assembly = "hg18", treatment = c(1, 1, 1, 1, 1, 1, 0, 0, 0, 0, 0, 0))

MyObjSi50_A<- methRead(file.listSi50_A,
          sample.id=list ("9", "15", "27", "35", "71", "89",
```

"3", "41", "45", "47", "49", "105"),

assembly = "hg18", treatment = c(1, 1, 1, 1, 1, 1, 0, 0, 0, 0, 0, 0))

##To get methylation stats and coverage stats use:

getMethylationStats(MyObj165E_L[[1]], plot = F, both.strands = F)##to get percentiles and quartiles

getMethylationStats(MyObj165E_L[[2]], plot = T, both.strands = F)## to get histogram of frequency distribution of %CpG methylation

getCoverageStats(MyObj165E_L[[1]], plot = T, both.strands = F)## to get histogram of CpG coverage per sample, change [[x]] to get coverage from different samples

getCoverageStats(MyObj165E_L[[2]], plot = T, both.strands = F)

#Filter CpG sites covered for at least 8 reads

filtered.myobj165E_L<-filterByCoverage(MyObj165E_L, lo.count = 8, lo.perc = NULL, hi.count = NULL, hi.perc = 99.9)

filtered.myobj165E_A<-filterByCoverage(MyObj165E_A, lo.count = 8, lo.perc = NULL, hi.count = NULL, hi.perc = 99.9)

filtered.myobjJi75_L<-filterByCoverage(MyObjJi75_L, lo.count = 8, lo.perc = NULL, hi.count = NULL, hi.perc = 99.9)

filtered.myobjJi75_A<-filterByCoverage(MyObjJi75_A, lo.count = 8, lo.perc = NULL, hi.count = NULL, hi.perc = 99.9)

filtered.myobjJi98_L<-filterByCoverage(MyObjJi98_L, lo.count = 8, lo.perc = NULL, hi.count = NULL, hi.perc = 99.9)

filtered.myobjJi98_A<-filterByCoverage(MyObjJi98_A, lo.count = 8, lo.perc = NULL, hi.count = NULL, hi.perc = 99.9)

filtered.myobjSi50_A<-filterByCoverage(MyObjSi50_A, lo.count = 8, lo.perc = NULL, hi.count = NULL, hi.perc = 99.9)

#We need to get the bases covered in all samples, the function unite will merge all samples to one object

Meth165E_L<-unite(filtered.myobj165E_L)

```
Meth165E_A<-unite(filtered.myobj165E_A)
MethJi75_L<-unite(filtered.myobjJi75_L)
MethJi75_A<-unite(filtered.myobjJi75_A)
MethJi98_L<-unite(filtered.myobjJi98_L)
MethJi98_A<-unite(filtered.myobjJi98_A)
MethSi50_A<-unite(filtered.myobjSi50_A)

#We can do PCA on our samples

ACPJi75L<-PCASamples(MethJi75_L, screeplot = FALSE,
            adj.lim = c(4e-04, 0.1), scale = TRUE, center = TRUE, comp = c(1, 2),
            transpose = FALSE, sd.filter = TRUE, sd.threshold = 0.5,
            filterByQuantile = TRUE, obj.return = TRUE, chunk.size = 1e+06)

ACP165EL<-PCASamples(Meth165E_L, screeplot = FALSE, transpose = TRUE , obj.return = TRUE)
ACP165EA<-PCASamples(Meth165E_A, screeplot = FALSE, transpose = TRUE , obj.return = TRUE)

ACPJi75L<-PCASamples(MethJi75_L, screeplot = TRUE, transpose = TRUE , obj.return = TRUE)
ACPJi75A<-PCASamples(MethJi75_A, screeplot = TRUE, transpose = TRUE , obj.return = TRUE)

ACPJi98L<-PCASamples(MethJi98_L, screeplot = FALSE, transpose = TRUE , obj.return = TRUE)
ACPJi98A<-PCASamples(MethJi98_A, screeplot = FALSE, transpose = TRUE , obj.return = TRUE)

ACPSi50A<-PCASamples(MethSi50_A, screeplot = FALSE, transpose = TRUE , obj.return = TRUE)

PCASamples(MethJi75_A)
PCASamples(MethJi98_L)
PCASamples(MethJi98_A)
PCASamples(MethSi50_A)

#The PCA coordinates are in column "x":
```

```
coord165E_A<-as.matrix(ACP165EA[["x"]])
coord165E_L<-as.matrix(ACP165EL[["x"]])
coordJi75_L<-as.matrix(ACPJi75L[["x"]])
coordJi75_A<-as.matrix(ACPJi75A[["x"]])
coordJi98_L<-as.matrix(ACPJi98L[["x"]])
coordJi98_A<-as.matrix(ACPJi98A[["x"]])
coordSi50_A<-as.matrix(ACPSi50A[["x"]])

#To graph PCA in a different way you can pick different variables: cos2 or contrib, etc.:

fvizJi75L<-fviz_eig(ACPJi75L)
indJi75L<-fviz_pca_ind(ACPJi75A,
        col.ind = "cos2", # Colorer par le cos2
        gradient.cols = c("#00AFBB", "#E7B800", "#FC4E07"),
        repel = TRUE)

fviz_pca_var(ACPJi75L, col.var = "contrib",
        gradient.cols = c("#00AFBB", "#E7B800", "#FC4E07"),
        repel = TRUE)

# Obtain Eigenvalues and contribution to variance by component
library(factoextra)
eig.val165A<- get_eigenvalue(ACP165EA)
eig.val165L<- get_eigenvalue(ACP165EL)

eig.valJi75A<- get_eigenvalue(ACPJi75A)
eig.valJi75L<- get_eigenvalue(ACPJi75L)

eig.valJi98A<- get_eigenvalue(ACPJi98A)
eig.valJi98L<- get_eigenvalue(ACPJi98L)
```

```
eig.valSi50A<- get_eigenvalue(ACPSi50A)


# Obtain variable results
res.var <- get_pca_var(ACPSi50A)
res.var$coord          # Coordinates
res.var$contrib        # Contributions to axes
res.var$cos2           # Quality of representation
#Obtain the contribution of variables to the axes of the PCA::
coordvar165E_L<-as.matrix(res.var[["contrib"]])
coontribvar165_A<-as.matrix(res.var[["contrib"]])
contribvarJi75_L<-as.matrix(res.var[["contrib"]])
contribvarJi75_A<-as.matrix(res.var[["contrib"]])
contribvarJi98_L<-as.matrix(res.var[["contrib"]])
contribvarJi98_A<-as.matrix(res.var[["contrib"]])
contribvarSi50_A<-as.matrix(res.var[["contrib"]])
# Individual results
res.ind <- get_pca_ind(ACPJi75A)
res.ind$coord          # Coordinates
res.ind$contrib        # Contributions to axes
res.ind$cos2           # Quality of representation


#If you want to save the matrix in a csv format
library(MASS)
write.csv(Your DataFrame,"Path where you'd like to export the DataFrame/File Name.csv", row.names = FALSE)
write.matrix(coord165E_A, file = "Coordonnees_ACP_165E_Apex.csv", sep = ";")
write.matrix(coord165E_L, file = "Coordonnees_ACP_165E_Leaves.csv", sep = ";")
write.matrix(eig.val165A, file = "Contributions_par_composant_165E_Apex.csv", sep = ";")
write.matrix(eig.val165L, file = "Contributions_par_composant_165E_Leaves.csv", sep = ";")
write.matrix(coordvar165E_L, file = "Contribution_des_variables_aux_axes_165E_Leaves.csv", sep = ";")
```

```
write.matrix(coontribvar165_A, file = "Contribution_des_variables_aux_axes_165E_Apex.csv", sep =
";")


write.matrix(coordJi75_A, file = "Coordonnees_ACP_Ji75_Apex.csv", sep = ";")

write.matrix(coordJi75_L, file = "Coordonnees_ACP_Ji75_Leaves.csv", sep = ";")

write.matrix(eig.valJi75A, file = "Contributions_par_composant_Ji75_Apex.csv", sep = ";")

write.matrix(eig.valJi75L, file = "Contributions_par_composant_Ji75_Leaves.csv", sep = ";")

write.matrix(contribvarJi75_A, file = "Contributions_des_variables_aux_axes_Ji75_Apex.csv", sep = ";")

write.matrix(contribvarJi75_L, file = "Contributions_des_variables_aux_axes_Ji75_Leaves.csv", sep =
";")


write.matrix(coordJi98_A, file = "Coordonnees_ACP_Ji98_Apex.csv", sep = ";")

write.matrix(coordJi98_L, file = "Coordonnees_ACP_Ji98_Leaves.csv", sep = ";")

write.matrix(eig.valJi98A, file = "Contributions_par_composant_Ji98_Apex.csv", sep = ";")

write.matrix(eig.valJi98L, file = "Contributions_par_composant_Ji98_Leaves.csv", sep = ";")

write.matrix(contribvarJi98_A, file = "Contributions_des_variables_aux_axes_Ji98_Apex.csv", sep = ";")

write.matrix(contribvarJi98_L, file = "Contributions_des_variables_aux_axes_Ji98_Leaves.csv", sep =
";")


write.matrix(coordSi50_A, file = "Coordonnees_ACP_Si50_Apex.csv", sep = ";")

write.matrix(eig.valSi50A, file = "Contributions_par_composant_Si50_Apex.csv", sep = ";")

write.matrix(contribvarSi50_A, file = "Contributions_des_variables_aux_axes_Si50_Apex.csv", sep =
";")


#Calculate Differential methylation between treatments (light vs shadow):

myDiff165E_L<- calculateDiffMeth(Meth165E_L)

MyDiff165EL.hyper<- getMethylDiff(myDiff165E_L, difference= 25, qvalue= 0.01, type= "hyper")

MyDiff165EL.hypo<- getMethylDiff(myDiff165E_L, difference= 25, qvalue= 0.01, type= "hypo")

myDiff165E_A<- calculateDiffMeth(Meth165E_A)

MyDiff165EA.hyper<- getMethylDiff(myDiff165E_A, difference= 25, qvalue= 0.01, type= "hyper")

MyDiff165EA.hypo<- getMethylDiff(myDiff165E_A, difference= 25, qvalue= 0.01, type= "hypo")
```

```
myDiffJi75_L<- calculateDiffMeth(MethJi75_L)
MyDiffJi75L.hyper<- getMethylDiff(myDiffJi75_L, difference= 25, qvalue= 0.01, type= "hyper")
MyDiffJi75L.hypo<- getMethylDiff(myDiffJi75_L, difference= 25, qvalue= 0.01, type= "hypo")
myDiffJi75_A<- calculateDiffMeth(MethJi75_A)
MyDiffJi75A.hyper<- getMethylDiff(myDiffJi75_A, difference= 25, qvalue= 0.01, type= "hyper")
MyDiffJi75A.hypo<- getMethylDiff(myDiffJi75_A, difference= 25, qvalue= 0.01, type= "hypo")

myDiffJi98_L<- calculateDiffMeth(MethJi98_L)
MyDiffJi98L.hypo<- getMethylDiff(myDiffJi98_L, difference= 25, qvalue= 0.01, type= "hypo")
MyDiffJi98L.hyper<- getMethylDiff(myDiffJi98_L, difference= 25, qvalue= 0.01, type= "hyper")
myDiffJi98_A<- calculateDiffMeth(MethJi98_A)
MyDiffJi98A.hyper<- getMethylDiff(myDiffJi98_A, difference= 25, qvalue= 0.01, type= "hyper")
MyDiffJi98A.hypo<- getMethylDiff(myDiffJi98_A, difference= 25, qvalue= 0.01, type= "hypo")

myDiffSi50_A<- calculateDiffMeth(MethSi50_A)
MyDiffSi50A.hyper<- getMethylDiff(myDiffSi50_A, difference= 25, qvalue= 0.01, type= "hyper")
MyDiffSi50A.hypo<- getMethylDiff(myDiffSi50_A, difference= 25, qvalue= 0.01, type= "hypo")

#Graph of hypo and hypermethylation per chromosome:
diffMethPerChr(myDiff165E_L, plot= TRUE, qvalue.cutoff = 0.01, meth.cutoff = 25)
diffMethPerChr(myDiff165E_A, plot= TRUE, qvalue.cutoff = 0.01, meth.cutoff = 25)
diffMethPerChr(myDiffJi75_L, plot= TRUE, qvalue.cutoff = 0.01, meth.cutoff = 25)
diffMethPerChr(myDiffJi75_A, plot= TRUE, qvalue.cutoff = 0.01, meth.cutoff = 25)
diffMethPerChr(myDiffJi98_L, plot= TRUE, qvalue.cutoff = 0.01, meth.cutoff = 25)
diffMethPerChr(myDiffJi98_A, plot= TRUE, qvalue.cutoff = 0.01, meth.cutoff = 25)
diffMethPerChr(myDiffSi50_A, plot= TRUE, qvalue.cutoff = 0.01, meth.cutoff = 25)
```

R SCRIPT: PCA _analysis CHH_CHG_ contexts.R

```
#PCA analysis from BSMAP methylation output files

#Read BSMAP methylation output files

#165E_Leaves
L165E_L_54_light<-read.table("BSMAP_methylation_output_54_light.tabular", sep="\t", header=TRUE)
L165E_L_40_light<-read.table("BSMAP_methylation_output_40_light.tabular", sep="\t", header=TRUE)
L165E_L_2_light<-read.table("BSMAP_methylation_output_2_light.tabular", sep="\t", header=TRUE)
L165E_L_26_light<-read.table("BSMAP_methylation_output_26_light.tabular", sep="\t", header=TRUE)
L165E_L_126_light<-read.table("BSMAP_methylation_output_126_light.tabular", sep="\t", header=TRUE)
L165E_L_8_light<-read.table("BSMAP_methylation_output_8_light.bed", sep="\t", header=TRUE)
L165E_L_62_shade<-read.table("BSMAP_methylation_output_62_shade.tabular", sep="\t", header=TRUE)
L165E_L_68_shade<-read.table("BSMAP_methylation_output_68_shade.tabular", sep="\t", header=TRUE)
L165E_L_78_shade<-read.table("BSMAP_methylation_output_78_shade.tabular", sep="\t", header=TRUE)
L165E_L_110_shade<-read.table("BSMAP_methylation_output_110_shade.bed", sep="\t", header=TRUE)
L165E_L_116_shade<-read.table("BSMAP_methylation_output_116_shade.bed", sep="\t", header=TRUE)
L165E_L_128_shade<-read.table("BSMAP_methylation_output_128_shade.tabular", sep="\t", header=TRUE)
#165E_Apex
L165E_A_1_light<-read.table("BSMAP_methylation_output_1_light.tabular", sep="\t", header=TRUE)
L165E_A_7_light<-read.table("BSMAP_methylation_output_7_light.tabular", sep="\t", header=TRUE)
L165E_A_25_light<-read.table("BSMAP_methylation_output_25_light.tabular", sep="\t", header=TRUE)
```

```
L165E_A_39_light<-read.table("BSMAP_methylation_output_39_light.tabular", sep="\t", header=TRUE)

L165E_A_53_light<-read.table("BSMAP_methylation_output_125_light.tabular", sep="\t", header=TRUE)

L165E_A_125_light<-read.table("BSMAP_methylation_output_8_light.bed", sep="\t", header=TRUE)

L165E_A_61_shade<-read.table("BSMAP_methylation_output_61_shade.tabular", sep="\t", header=TRUE)

L165E_A_67_shade<-read.table("BSMAP_methylation_output_67_shade.tabular", sep="\t", header=TRUE)

L165E_A_77_shade<-read.table("BSMAP_methylation_output_77_shade.tabular", sep="\t", header=TRUE)

L165E_A_109_shade<-read.table("BSMAP_methylation_output_109_shade.bed", sep="\t", header=TRUE)

L165E_A_115_shade<-read.table("BSMAP_methylation_output_115_shade.tabular", sep="\t", header=TRUE)

L165E_A_127_shade<-read.table("BSMAP_methylation_output_127_shade.tabular", sep="\t", header=TRUE)

setwd("~/Line _Ji75_CHH_CHG")

#Ji75_Leaves

Ji75_L_34_light<-read.table("BSMAP_methylation_output_34_light.tabular", sep="\t", header=TRUE)

Ji75_L_64_light<-read.table("BSMAP_methylation_output_64_light.tabular", sep="\t", header=TRUE)

Ji75_L_76_light<-read.table("BSMAP_methylation_output_76_light.tabular", sep="\t", header=TRUE)

Ji75_L_84_light<-read.table("BSMAP_methylation_output_84_light.tabular", sep="\t", header=TRUE)

Ji75_L_112_light<-read.table("BSMAP_methylation_output_112_light.bed", sep="\t", header=TRUE)

Ji75_L_136_light<-read.table("BSMAP_methylation_output_136_light.tabular", sep="\t", header=TRUE)

Ji75_L_60_shade<-read.table("BSMAP_methylation_output_60_shade.tabular", sep="\t", header=TRUE)

Ji75_L_80_shade<-read.table("BSMAP_methylation_output_80_shade.tabular", sep="\t", header=TRUE)

Ji75_L_86_shade<-read.table("BSMAP_methylation_output_86_shade.tabular", sep="\t", header=TRUE)

Ji75_L_102_shade<-read.table("BSMAP_methylation_output_102_shade.bed", sep="\t", header=TRUE)

Ji75_L_120_shade<-read.table("BSMAP_methylation_output_120_shade.bed", sep="\t", header=TRUE)
```

```
Ji75_L_122_shade<-read.table("BSMAP_methylation_output_122_shade.tabular", sep="\t", header=TRUE)

#Ji75_Apex

Ji75_A_33_light<-read.table("BSMAP_methylation_output_33_light.tabular", sep="\t", header=TRUE)

Ji75_A_63_light<-read.table("BSMAP_methylation_output_63_light.tabular", sep="\t", header=TRUE)

Ji75_A_75_light<-read.table("BSMAP_methylation_output_75_light.tabular", sep="\t", header=TRUE)

Ji75_A_83_light<-read.table("BSMAP_methylation_output_83_light.tabular", sep="\t", header=TRUE)

Ji75_A_111_light<-read.table("BSMAP_methylation_output_111_light.bed", sep="\t", header=TRUE)

Ji75_A_135_light<-read.table("BSMAP_methylation_output_135_light.tabular", sep="\t", header=TRUE)

Ji75_A_59_shade<-read.table("BSMAP_methylation_output_59_shade.tabular", sep="\t", header=TRUE)

Ji75_A_79_shade<-read.table("BSMAP_methylation_output_79_shade.tabular", sep="\t", header=TRUE)

Ji75_A_85_shade<-read.table("BSMAP_methylation_output_85_shade.tabular", sep="\t", header=TRUE)

Ji75_A_101_shade<-read.table("BSMAP_methylation_output_101_shade.bed", sep="\t", header=TRUE)

Ji75_A_119_shade<-read.table("BSMAP_methylation_output_119_shade.bed", sep="\t", header=TRUE)

Ji75_A_121_shade<-read.table("BSMAP_methylation_output_121_shade.tabular", sep="\t", header=TRUE)

#Ji98_Leaves

Ji98_L_38_light<-read.table("BSMAP_methylation_output_38_light.tabular", sep="\t", header=TRUE)

Ji98_L_56_light<-read.table("BSMAP_methylation_output_56_light.tabular", sep="\t", header=TRUE)

Ji98_L_66_light<-read.table("BSMAP_methylation_output_66_light.tabular", sep="\t", header=TRUE)

Ji98_L_74_light<-read.table("BSMAP_methylation_output_74_light.tabular", sep="\t", header=TRUE)

Ji98_L_114_light<-read.table("BSMAP_methylation_output_114_light.bed", sep="\t", header=TRUE)

Ji98_L_132_light<-read.table("BSMAP_methylation_output_132_light.tabular", sep="\t", header=TRUE)

Ji98_L_44_shade<-read.table("BSMAP_methylation_output_44_shade.tabular", sep="\t", header=TRUE)

Ji98_L_52_shade<-read.table("BSMAP_methylation_output_52_shade.tabular", sep="\t", header=TRUE)

Ji98_L_92_shade<-read.table("BSMAP_methylation_output_92_shade.bed", sep="\t", header=TRUE)
```

```
Ji98_L_100_shade<-read.table("BSMAP_methylation_output_100_shade.bed", sep="\t", header=TRUE)

Ji98_L_108_shade<-read.table("BSMAP_methylation_output_108_shade.bed", sep="\t", header=TRUE)

Ji98_L_124_shade<-read.table("BSMAP_methylation_output_124_shade.tabular", sep="\t", header=TRUE)

#Ji98_Apex

Ji98_A_13_light<-read.table("BSMAP_methylation_output_13_light.tabular", sep="\t", header=TRUE)

Ji98_A_55_light<-read.table("BSMAP_methylation_output_55_light.tabular", sep="\t", header=TRUE)

Ji98_A_65_light<-read.table("BSMAP_methylation_output_65_light.tabular", sep="\t", header=TRUE)

Ji98_A_73_light<-read.table("BSMAP_methylation_output_73_light.tabular", sep="\t", header=TRUE)

Ji98_A_113_light<-read.table("BSMAP_methylation_output_113_light.bed", sep="\t", header=TRUE)

Ji98_A_131_light<-read.table("BSMAP_methylation_output_131_light.tabular", sep="\t", header=TRUE)

Ji98_A_43_shade<-read.table("BSMAP_methylation_output_43_shade.tabular", sep="\t", header=TRUE)

Ji98_A_51_shade<-read.table("BSMAP_methylation_output_51_shade.tabular", sep="\t", header=TRUE)

Ji98_A_91_shade<-read.table("BSMAP_methylation_output_91_shade.bed", sep="\t", header=TRUE)

Ji98_A_99_shade<-read.table("BSMAP_methylation_output_99_shade.bed", sep="\t", header=TRUE)

Ji98_A_107_shade<-read.table("BSMAP_methylation_output_107_shade.bed", sep="\t", header=TRUE)

Ji98_A_123_shade<-read.table("BSMAP_methylation_output_123_shade.tabular", sep="\t", header=TRUE)

#Si50_Apex

Si50_A_9_light<-read.table("BSMAP_methylation_output_9_light.bed", sep="\t", header=TRUE)

Si50_A_15_light<-read.table("BSMAP_methylation_output_15_light.tabular", sep="\t", header=TRUE)

Si50_A_27_light<-read.table("BSMAP_methylation_output_27_light.tabular", sep="\t", header=TRUE)

Si50_A_35_light<-read.table("BSMAP_methylation_output_35_light.tabular", sep="\t", header=TRUE)

Si50_A_71_light<-read.table("BSMAP_methylation_output_71_light.tabular", sep="\t", header=TRUE)

Si50_A_89_light<-read.table("BSMAP_methylation_output_89_light.bed", sep="\t", header=TRUE)

Si50_A_3_shade<-read.table("BSMAP_methylation_output_3_shade.tabular", sep="\t", header=TRUE)

Si50_A_41_shade<-read.table("BSMAP_methylation_output_41_shade.tabular", sep="\t", header=TRUE)
```

```
Si50_A_45_shade<-read.table("BSMAP_methylation_output_45_shade.tabular", sep="\t", header=TRUE)

Si50_A_47_shade<-read.table("BSMAP_methylation_output_47_shade.tabular", sep="\t", header=TRUE)

Si50_A_49_shade<-read.table("BSMAP_methylation_output_49_shade.tabular", sep="\t", header=TRUE)

Si50_A_105_shade<-read.table("BSMAP_methylation_output_105_shade.bed", sep="\t", header=TRUE)


#Filter "CHG" context from BSMAP_methylation_output files, chose the cytosines covered by 7 or more reads:

library(tidyverse)

##Filter "CHG" context from Line 165E

L165E_L_54_light2<- L165E_L_54_light[,c(1,2,4,5,7)]

L165E_L_54_light_CHG<-filter(L165E_L_54_light2, context=="CHG" & C_count>7)

L165E_L54<-L165E_L_54_light_CHG[,c(1,2,4)] %>%

  rename("54_light" = ratio)

L165E_L_2_light2<- L165E_L_2_light[,c(1,2,4,5,7)]

L165E_L_2_light_CHG<-filter(L165E_L_2_light2, context=="CHG" & C_count>7)

L165E_L2<-L165E_L_2_light_CHG[,c(1,2,4)] %>%

  rename("2_light" = ratio)

L165E_L_26_light2<- L165E_L_26_light[,c(1,2,4,5,7)]

L165E_L_26_light_CHG<-filter(L165E_L_26_light2, context=="CHG" & C_count>7)

L165E_L26<-L165E_L_26_light_CHG[,c(1,2,4)] %>%

  rename("26_light" = ratio)

L165E_L_40_light2<- L165E_L_40_light[,c(1,2,4,5,7)]

L165E_L_40_light_CHG<-filter(L165E_L_40_light2, context=="CHG" & C_count>7)

L165E_L40<-L165E_L_40_light_CHG[,c(1,2,4)] %>%

  rename("40_light" = ratio)

L165E_L_8_light2<- L165E_L_8_light[,c(1,2,4,5,7)]

L165E_L_8_light_CHG<-filter(L165E_L_8_light2, context=="CHG" & C_count>7)

L165E_L8<-L165E_L_8_light_CHG[,c(1,2,4)] %>%

  rename("8_light" = ratio)
```

```
L165E_L_126_light2<- L165E_L_126_light[,c(1,2,4,5,7)]
L165E_L_126_light_CHG<-filter(L165E_L_126_light2, context=="CHG" & C_count>7)
L165E_L126<-L165E_L_126_light_CHG[,c(1,2,4)] %>%
  rename("126_light" = ratio)
L165E_L_62_shade2<- L165E_L_62_shade[,c(1,2,4,5,7)]
L165E_L_62_shade_CHG<-filter(L165E_L_62_shade2, context=="CHG" & C_count>7)
L165E_L62<-L165E_L_62_shade_CHG[,c(1,2,4)] %>%
  rename("62_shade" = ratio)
L165E_L_68_shade2<- L165E_L_68_shade[,c(1,2,4,5,7)]
L165E_L_68_shade_CHG<-filter(L165E_L_68_shade2, context=="CHG" & C_count>7)
L165E_L68<-L165E_L_68_shade_CHG[,c(1,2,4)] %>%
  rename("68_shade" = ratio)
L165E_L_78_shade2<- L165E_L_78_shade[,c(1,2,4,5,7)]
L165E_L_78_shade_CHG<-filter(L165E_L_78_shade2, context=="CHG" & C_count>7)
L165E_L78<-L165E_L_78_shade_CHG[,c(1,2,4)] %>%
  rename("78_light" = ratio)
L165E_L_110_shade2<- L165E_L_110_shade[,c(1,2,4,5,7)]
L165E_L_110_shade_CHG<-filter(L165E_L_110_shade2, context=="CHG" & C_count>7)
L165E_L110<-L165E_L_110_shade_CHG[,c(1,2,4)] %>%
  rename("110_shade" = ratio)
L165E_L_116_shade2<- L165E_L_116_shade[,c(1,2,4,5,7)]
L165E_L_116_shade_CHG<-filter(L165E_L_116_shade2, context=="CHG" & C_count>7)
L165E_L116<-L165E_L_116_shade_CHG[,c(1,2,4)] %>%
  rename("116_shade" = ratio)
L165E_L_128_shade2<- L165E_L_128_shade[,c(1,2,4,5,7)]
L165E_L_128_shade_CHG<-filter(L165E_L_128_shade2, context=="CHG" & C_count>7)
L165E_L128<-L165E_L_128_shade_CHG[,c(1,2,4)] %>%
  rename("128_shade" = ratio)
L165E_A_1_light2<- L165E_A_1_light[,c(1,2,4,5,7)]
L165E_A_1_light_CHG<-filter(L165E_A_1_light2, context=="CHG" & C_count>7)
L165E_A1<-L165E_A_1_light_CHG[,c(1,2,4)] %>%
```

```
  rename("1_light" = ratio)
L165E_A_7_light2<- L165E_A_7_light[,c(1,2,4,5,7)]
L165E_A_7_light_CHG<-filter(L165E_A_7_light2, context=="CHG" & C_count>7)
#Filter "CHG" context from Line Ji75
Ji75_A_33_light2<- Ji75_A_33_light[,c(1,2,4,5,7)]
Ji75_A_33_light_CHG<-filter(Ji75_A_33_light2, context=="CHG" & C_count>7)
Ji75_A33<-Ji75_A_33_light_CHG[,c(1,2,4)] %>%
  rename("33_light" = ratio)
Ji75_A_63_light2<- Ji75_A_63_light[,c(1,2,4,5,7)]
Ji75_A_63_light_CHG<-filter(Ji75_A_63_light2, context=="CHG" & C_count>7)
Ji75_A63<-Ji75_A_63_light_CHG[,c(1,2,4)] %>%
  rename("63_light" = ratio)
Ji75_A_75_light2<- Ji75_A_75_light[,c(1,2,4,5,7)]
Ji75_A_75_light_CHG<-filter(Ji75_A_75_light2, context=="CHG" & C_count>7)
Ji75_A75<-Ji75_A_75_light_CHG[,c(1,2,4)] %>%
  rename("75_light" = ratio)
Ji75_A_83_light2<- Ji75_A_83_light[,c(1,2,4,5,7)]
Ji75_A_83_light_CHG<-filter(Ji75_A_83_light2, context=="CHG" & C_count>7)
Ji75_A83<-Ji75_A_83_light_CHG[,c(1,2,4)] %>%
  rename("83_light" = ratio)
Ji75_A_111_light2<- Ji75_A_111_light[,c(1,2,4,5,7)]
Ji75_A_111_light_CHG<-filter(Ji75_A_111_light2, context=="CHG" & C_count>7)
Ji75_A111<-Ji75_A_111_light_CHG[,c(1,2,4)] %>%
  rename("111_light" = ratio)
Ji75_A_135_light2<- Ji75_A_135_light[,c(1,2,4,5,7)]
Ji75_A_135_light_CHG<-filter(Ji75_A_135_light2, context=="CHG" & C_count>7)
Ji75_A135<-Ji75_A_135_light_CHG[,c(1,2,4)] %>%
  rename("135_light" = ratio)
Ji75_A_59_shade2<- Ji75_A_59_shade[,c(1,2,4,5,7)]
Ji75_A_59_shade_CHG<-filter(Ji75_A_59_shade2, context=="CHG" & C_count>7)
Ji75_A59<-Ji75_A_59_shade_CHG[,c(1,2,4)] %>%
```

```
  rename("59_shade" = ratio)
Ji75_A_79_shade2<- Ji75_A_79_shade[,c(1,2,4,5,7)]
Ji75_A_79_shade_CHG<-filter(Ji75_A_79_shade2, context=="CHG" & C_count>7)
Ji75_A79<-Ji75_A_79_shade_CHG[,c(1,2,4)] %>%
  rename("79_shade" = ratio)
Ji75_A_85_shade2<- Ji75_A_85_shade[,c(1,2,4,5,7)]
Ji75_A_85_shade_CHG<-filter(Ji75_A_85_shade2, context=="CHG" & C_count>7)
Ji75_A85<-Ji75_A_85_shade_CHG[,c(1,2,4)] %>%
  rename("85_shade" = ratio)
Ji75_A_101_shade2<- Ji75_A_101_shade[,c(1,2,4,5,7)]
Ji75_A_101_shade_CHG<-filter(Ji75_A_101_shade2, context=="CHG" & C_count>7)
Ji75_A101<-Ji75_A_101_shade_CHG[,c(1,2,4)] %>%
  rename("101_shade" = ratio)
Ji75_A_119_shade2<- Ji75_A_119_shade[,c(1,2,4,5,7)]
Ji75_A_119_shade_CHG<-filter(Ji75_A_119_shade2, context=="CHG" & C_count>7)
Ji75_A119<-Ji75_A_119_shade_CHG[,c(1,2,4)] %>%
  rename("119_shade" = ratio)
Ji75_A_121_shade2<- Ji75_A_121_shade[,c(1,2,4,5,7)]
Ji75_A_121_shade_CHG<-filter(Ji75_A_121_shade2, context=="CHG" & C_count>7)
Ji75_A121<-Ji75_A_121_shade_CHG[,c(1,2,4)] %>%
  rename("121_shade" = ratio)

#Filter "CHG" context for Line Ji98
Ji98_A_55_light2<- Ji98_A_55_light[,c(1,2,4,5,7)]
Ji98_A55_light_CHG<-filter(Ji98_A_55_light2, context=="CHG" & C_count>7)
Ji98_A55<- Ji98_A55_light_CHG[,c(1,2,4)] %>%
rename("ratio_55A" = ratio)
Ji98_A_65_light2<- Ji98_A_65_light[,c(1,2,4,5,7)]
Ji98_A65_light_CHG<-filter(Ji98_A_65_light2, context=="CHG" & C_count>7)
Ji98_A65<- Ji98_A65_light_CHG[,c(1,2,4)] %>%
rename("ratio_65A" = ratio)
```

```
Ji98_A_73_light2<- Ji98_A_73_light[,c(1,2,4,5,7)]
Ji98_A73_light_CHG<-filter(Ji98_A_73_light2, context=="CHG" & C_count>7)
Ji98_A73<- Ji98_A73_light_CHG[,c(1,2,4)] %>%
rename("ratio_73A" = ratio)
Ji98_A_113_light2<- Ji98_A_113_light[,c(1,2,4,5,7)]
Ji98_A113_light_CHG<-filter(Ji98_A_113_light2, context=="CHG" & C_count>7)
Ji98_A113<- Ji98_A113_light_CHG[,c(1,2,4)] %>%
rename("ratio_113A" = ratio)
Ji98_A_131_light2<- Ji98_A_131_light[,c(1,2,4,5,7)]
Ji98_A131_light_CHG<-filter(Ji98_A_131_light2, context=="CHG" & C_count>7)
Ji98_A131<- Ji98_A131_light_CHG[,c(1,2,4)] %>%
rename("ratio_131A" = ratio)

Ji98_A_43_shade2<- Ji98_A_43_shade[,c(1,2,4,5,7)]
Ji98_A43_shade_CHG<-filter(Ji98_A_43_shade2, context=="CHG" & C_count>7)
Ji98_A43<- Ji98_A43_shade_CHG[,c(1,2,4)] %>%
rename("ratio_43A" = ratio)
Ji98_A_51_shade2<- Ji98_A_51_shade[,c(1,2,4,5,7)]
Ji98_A51_shade_CHG<-filter(Ji98_A_51_shade2, context=="CHG" & C_count>7)
Ji98_A51<- Ji98_A51_shade_CHG[,c(1,2,4)] %>%
rename("ratio_51A" = ratio)
Ji98_A_91_shade2<- Ji98_A_91_shade[,c(1,2,4,5,7)]
Ji98_A91_shade_CHG<-filter(Ji98_A_91_shade2, context=="CHG" & C_count>7)
Ji98_A91<- Ji98_A91_shade_CHG[,c(1,2,4)] %>%
rename("ratio_91A" = ratio)
Ji98_A_99_shade2<- Ji98_A_99_shade[,c(1,2,4,5,7)]
Ji98_A99_shade_CHG<-filter(Ji98_A_99_shade2, context=="CHG" & C_count>7)
Ji98_A99<- Ji98_A99_shade_CHG[,c(1,2,4)] %>%
rename("ratio_99A" = ratio)
Ji98_A_107_shade2<- Ji98_A_107_shade[,c(1,2,4,5,7)]
Ji98_A107_shade_CHG<-filter(Ji98_A_107_shade2, context=="CHG" & C_count>7)
```

```
Ji98_A107<- Ji98_A107_shade_CHG[,c(1,2,4)] %>%
rename("ratio_107A" = ratio)
Ji98_A_123_shade2<- Ji98_A_123_shade[,c(1,2,4,5,7)]
Ji98_A123_shade_CHG<-filter(Ji98_A_123_shade2, context=="CHG" & C_count>7)
Ji98_A123<- Ji98_A123_shade_CHG[,c(1,2,4)] %>%
  rename("ratio_123A" = ratio)
#Filter "CHG" context for Line Si50


Si50_A_9_light2<- Si50_A_9_light[,c(1,2,4,5,7)]
Si50_A_9_light_CHG<-filter(Si50_A_9_light2, context=="CHG" & C_count>7)
Si50_A9<-Si50_A_9_light_CHG[,c(1,2,4)] %>%
 rename("9_light" = ratio)
Si50_A_15_light2<- Si50_A_15_light[,c(1,2,4,5,7)]
Si50_A_15_light_CHG<-filter(Si50_A_15_light2, context=="CHG" & C_count>7)
Si50_A15<-Si50_A_15_light_CHG[,c(1,2,4)] %>%
 rename("15_light" = ratio)
Si50_A_27_light2<- Si50_A_27_light[,c(1,2,4,5,7)]
Si50_A_27_light_CHG<-filter(Si50_A_27_light2, context=="Change" & C_count>7)
Si50_A27<-Si50_A_27_light_CHG[,c(1,2,4)] %>%
  rename("27_light" = ratio)
Si50_A_35_light2<- Si50_A_35_light[,c(1,2,4,5,7)]
Si50_A_35_light_CHG<-filter(Si50_A_35_light2, context=="CHG" & C_count>7)
Si50_A35<-Si50_A_35_light_CHG[,c(1,2,4)] %>%
  rename("35_light" = ratio)
Si50_A_71_light2<- Si50_A_71_light[,c(1,2,4,5,7)]
Si50_A_71_light_CHG<-filter(Si50_A_71_light2, context=="CHG" & C_count>7)
Si50_A71<-Si50_A_71_light_CHG[,c(1,2,4)] %>%
  rename("71_light" = ratio)
Si50_A_89_light2<- Si50_A_89_light[,c(1,2,4,5,7)]
Si50_A_89_light_CHG<-filter(Si50_A_89_light2, context=="CHG" & C_count>7)
Si50_A89<-Si50_A_89_light_CHG[,c(1,2,4)] %>%
```

```
  rename("89_light" = ratio)

#Shade:

Si50_A_3_shade2<- Si50_A_3_shade[,c(1,2,4,5,7)]

Si50_A_3_shade_CHG<-filter(Si50_A_3_shade2, context=="CHG" & C_count>7)

Si50_A3<-Si50_A_3_shade_CHG[,c(1,2,4)] %>%

  rename("3_shade" = ratio)

Si50_A_41_shade2<- Si50_A_41_shade[,c(1,2,4,5,7)]

Si50_A_41_shade_CHG<-filter(Si50_A_41_shade2, context=="CHG" & C_count>7)

Si50_A41<-Si50_A_41_shade_CHG[,c(1,2,4)] %>%

  rename("41_shade" = ratio)

Si50_A_45_shade2<- Si50_A_45_shade[,c(1,2,4,5,7)]

Si50_A_45_shade_CHG<-filter(Si50_A_45_shade2, context=="CHG" & C_count>7)

Si50_A45<-Si50_A_45_shade_CHG[,c(1,2,4)] %>%

  rename("45_shade" = ratio)

Si50_A_47_shade2<- Si50_A_47_shade[,c(1,2,4,5,7)]

Si50_A_47_shade_CHG<-filter(Si50_A_47_shade2, context=="CHG" & C_count>7)

Si50_A47<-Si50_A_47_shade_CHG[,c(1,2,4)] %>%

  rename("47_shade" = ratio)

Si50_A_49_shade2<- Si50_A_49_shade[,c(1,2,4,5,7)]

Si50_A_49_shade_CHG<-filter(Si50_A_49_shade2, context=="CHG" & C_count>7)

Si50_A49<-Si50_A_49_shade_CHG[,c(1,2,4)] %>%

  rename("49_shade" = ratio)

Si50_A_105_shade2<- Si50_A_105_shade[,c(1,2,4,5,7)]

Si50_A_105_shade_CHG<-filter(Si50_A_105_shade2, context=="CHG" & C_count>7)

Si50_A105<-Si50_A_105_shade_CHG[,c(1,2,4)] %>%

  rename("105_shade" = ratio)


#Filter "CHH" context from BSMAP_methylation_output files, chose the cytosines covered by 7 or more reads:

##Filter "CHH" context from Line 165E

Ji75_L_34_light2<- Ji75_L_34_light[,c(1,2,4,5,7)]
```

```
Ji75_L_34_light_CHH<-filter(Ji75_L_34_light2, context=="CHH" & C_count>7)
Ji75_L34<-Ji75_L_34_light_CHH[,c(1,2,4)] %>%
  rename("34_light" = ratio)
Ji75_L_64_light2<- Ji75_L_64_light[,c(1,2,4,5,7)]
Ji75_L_64_light_CHH<-filter(Ji75_L_64_light2, context=="CHH" & C_count>7)
Ji75_L64<-Ji75_L_64_light_CHH[,c(1,2,4)] %>%
  rename("64_light" = ratio)
Ji75_L_76_light2<- Ji75_L_76_light[,c(1,2,4,5,7)]
Ji75_L_76_light_CHH<-filter(Ji75_L_76_light2, context=="CHH" & C_count>7)
Ji75_L76<-Ji75_L_76_light_CHH[,c(1,2,4)] %>%
  rename("76_light" = ratio)
Ji75_L_84_light2<- Ji75_L_84_light[,c(1,2,4,5,7)]
Ji75_L_84_light_CHH<-filter(Ji75_L_84_light2, context=="CHH" & C_count>7)
Ji75_L84<-Ji75_L_84_light_CHH[,c(1,2,4)] %>%
  rename("84_light" = ratio)
Ji75_L_112_light2<- Ji75_L_112_light[,c(1,2,4,5,7)]
Ji75_L_112_light_CHH<-filter(Ji75_L_112_light2, context=="CHH" & C_count>7)
Ji75_L112<-Ji75_L_112_light_CHH[,c(1,2,4)] %>%
  rename("112_light" = ratio)
Ji75_L_136_light2<- Ji75_L_136_light[,c(1,2,4,5,7)]
Ji75_L_136_light_CHH<-filter(Ji75_L_136_light2, context=="CHH" & C_count>7)
Ji75_L136<-Ji75_L_136_light_CHH[,c(1,2,4)] %>%
  rename("136_light" = ratio)
Ji75_L_84_light2<- Ji75_L_84_light[,c(1,2,4,5,7)]
Ji75_L_84_light_CHH<-filter(Ji75_L_84_light2, context=="CHH" & C_count>7)
Ji75_L84<-Ji75_L_84_light_CHH[,c(1,2,4)] %>%
  rename("84_light" = ratio)
Ji75_L_60_shade2<- Ji75_L_60_shade[,c(1,2,4,5,7)]
Ji75_L_60_shade_CHH<-filter(Ji75_L_60_shade2, context=="CHH" & C_count>7)
Ji75_L60<-Ji75_L_60_shade_CHH[,c(1,2,4)] %>%
  rename("60_shade" = ratio)
```

```
Ji75_L_80_shade2<- Ji75_L_80_shade[,c(1,2,4,5,7)]
Ji75_L_80_shade_CHH<-filter(Ji75_L_80_shade2, context=="CHH" & C_count>7)
Ji75_L80<-Ji75_L_80_shade_CHH[,c(1,2,4)] %>%
  rename("80_shade" = ratio)
Ji75_L_86_shade2<- Ji75_L_86_shade[,c(1,2,4,5,7)]
Ji75_L_86_shade_CHH<-filter(Ji75_L_86_shade2, context=="CHH" & C_count>7)
Ji75_L86<-Ji75_L_86_shade_CHH[,c(1,2,4)] %>%
  rename("86_shade" = ratio)
Ji75_L_102_shade2<- Ji75_L_102_shade[,c(1,2,4,5,7)]
Ji75_L_102_shade_CHH<-filter(Ji75_L_102_shade2, context=="CHH" & C_count>7)
Ji75_L102<-Ji75_L_102_shade_CHH[,c(1,2,4)] %>%
  rename("102_shade" = ratio)
Ji75_L_120_shade2<- Ji75_L_120_shade[,c(1,2,4,5,7)]
Ji75_L_120_shade_CHH<-filter(Ji75_L_120_shade2, context=="CHH" & C_count>7)
Ji75_L120<-Ji75_L_120_shade_CHH[,c(1,2,4)] %>%
  rename("120_shade" = ratio)
Ji75_L_122_shade2<- Ji75_L_122_shade[,c(1,2,4,5,7)]
Ji75_L_122_shade_CHH<-filter(Ji75_L_122_shade2, context=="CHH" & C_count>7)
Ji75_L122<-Ji75_L_122_shade_CHH[,c(1,2,4)] %>%
  rename("122_shade" = ratio)

##Filter "CHH" context from Line 165E

L165E_A7<-L165E_A_7_light_CHH[,c(1,2,4)] %>%
rename("7_light" = ratio)
L165E_A_25_light2<- L165E_A_25_light[,c(1,2,4,5,7)]
L165E_A_25_light_CHH<-filter(L165E_A_25_light2, context=="CHH" & C_count>7)
L165E_A25<-L165E_A_25_light_CHH[,c(1,2,4)] %>%
rename("25_light" = ratio)
L165E_A_39_light2<- L165E_A_39_light[,c(1,2,4,5,7)]
L165E_A_39_light_CHH<-filter(L165E_A_39_light2, context=="CHH" & C_count>7)
```

```
L165E_A39<-L165E_A_39_light_CHH[,c(1,2,4)] %>%
rename("39_light" = ratio)
L165E_A_53_light2<- L165E_A_53_light[,c(1,2,4,5,7)]
L165E_A_53_light_CHH<-filter(L165E_A_53_light2, context=="CHH" & C_count>7)
L165E_A53<-L165E_A_53_light_CHH[,c(1,2,4)] %>%
rename("53_light" = ratio)
L165E_A_125_light2<- L165E_A_125_light[,c(1,2,4,5,7)]
L165E_A_125_light_CHH<-filter(L165E_A_125_light2, context=="CHH" & C_count>7)
L165E_A125<-L165E_A_125_light_CHH[,c(1,2,4)] %>%
rename("125_light" = ratio)

#Filter "CHH" context from Line Ji98

Ji98_L_38_light2<- Ji98_L_38_light[,c(1,2,4, 5,7)]
Ji98_L38_light_CHH<-filter(Ji98_L_38_light2, context=="CHH" & C_count>7)
Ji98_L38<- Ji98_L38_light_CHH[,c(1,2,4)] %>%
  rename("ratio_38L" = ratio)
Ji98_L_56_light2<- Ji98_L_56_light[,c(1,2,4, 5,7)]
Ji98_L56_light_CHH<-filter(Ji98_L_56_light2, context=="CHH" & C_count>7)
Ji98_L56<- Ji98_L56_light_CHH[,c(1,2,4)] %>%
  rename("ratio_56L" = ratio)
Ji98_L_66_light2<- Ji98_L_66_light[,c(1,2,4, 5,7)]
Ji98_L66_light_CHH<-filter(Ji98_L_66_light2, context=="CHH" & C_count>7)
Ji98_L66<- Ji98_L66_light_CHH[,c(1,2,4)] %>%
  rename("ratio_66L" = ratio)
Ji98_L_74_light2<- Ji98_L_74_light[,c(1,2,4, 5,7)]
Ji98_L74_light_CHH<-filter(Ji98_L_74_light2, context=="CHH" & C_count>7)
Ji98_L74<- Ji98_L74_light_CHH[,c(1,2,4)] %>%
  rename("ratio_74L" = ratio)
Ji98_L_114_light2<-Ji98_L_114_light[,c(1,2,4,5,7)]
Ji98_L_114_light_CHH<-filter(Ji98_L_114_light2, context=="CHH" & C_count>7)
```

```
Ji98_L114<- Ji98_L_114_light_CHH[,c(1,2,4)] %>%
  rename("ratio_114L" = ratio)
Ji98_L_132_light2<-Ji98_L_132_light[,c(1,2,4,5,7)]
Ji98_L_132_light_CHH<-filter(Ji98_L_132_light2, context=="CHH" & C_count>7)
Ji98_L132<- Ji98_L_132_light_CHH[,c(1,2,4)] %>%
  rename("ratio_132L" = ratio)
Ji98_L_44_shade2<- Ji98_L_44_shade[,c(1,2,4, 5,7)]
Ji98_L44_shade_CHH<-filter(Ji98_L_44_shade2, context=="CHH" & C_count>7)
Ji98_L44<- Ji98_L44_shade_CHH[,c(1,2,4)] %>%
  rename("ratio_44L" = ratio)
Ji98_L_52_shade2<- Ji98_L_52_shade[,c(1,2,4, 5,7)]
Ji98_L52_shade_CHH<-filter(Ji98_L_52_shade2, context=="CHH" & C_count>7)
Ji98_L52<- Ji98_L52_shade_CHH[,c(1,2,4)] %>%
  rename("ratio_52L" = ratio)
Ji98_L_92_shade2<- Ji98_L_92_shade[,c(1,2,4, 5,7)]
Ji98_L92_shade_CHH<-filter(Ji98_L_92_shade2, context=="CHH" & C_count>7)
Ji98_L92<- Ji98_L92_shade_CHH[,c(1,2,4)] %>%
  rename("ratio_92L" = ratio)
Ji98_L_100_shade2<- Ji98_L_100_shade[,c(1,2,4, 5,7)]
Ji98_L100_shade_CHH<-filter(Ji98_L_100_shade2, context=="CHH" & C_count>7)
Ji98_L100<- Ji98_L100_shade_CHH[,c(1,2,4)] %>%
  rename("ratio_100L" = ratio)
Ji98_L_108_shade2<- Ji98_L_108_shade[,c(1,2,4, 5,7)]
Ji98_L108_shade_CHH<-filter(Ji98_L_108_shade2, context=="CHH" & C_count>7)
Ji98_L108<- Ji98_L108_shade_CHH[,c(1,2,4)] %>%
  rename("ratio_108L" = ratio)
Ji98_L_124_shade2<- Ji98_L_124_shade[,c(1,2,4, 5,7)]
Ji98_L124_shade_CHH<-filter(Ji98_L_124_shade2, context=="CHH" & C_count>7)
Ji98_L124<- Ji98_L124_shade_CHH[,c(1,2,4)] %>%
  rename("ratio_124L" = ratio)
#Filter "CHH" or "CHG" context from Line Ji98 Tissue Apex
```

```
Ji98_A_13_light2<- Ji98_A_13_light[,c(1,2,4,5,7)]
Ji98_A13_light_CHH<-filter(Ji98_A_13_light2, context=="CHH" & C_count>7)
Ji98_A13<- Ji98_A13_light_CHH[,c(1,2,4)] %>%
  rename("ratio_13A" = ratio)


#Filter "CHH" context from Line Si50
Si50_A_9_light2<- Si50_A_9_light[,c(1,2,4,5,7)]
Si50_A_9_light_CHH<-filter(Si50_A_9_light2, context=="CHH" & C_count>7)
Si50_A9<-Si50_A_9_light_CHH[,c(1,2,4)] %>%
  rename("9_light" = ratio)
Si50_A_15_light2<- Si50_A_15_light[,c(1,2,4,5,7)]
Si50_A_15_light_CHH<-filter(Si50_A_15_light2, context=="CHH" & C_count>7)
Si50_A15<-Si50_A_15_light_CHH[,c(1,2,4)] %>%
  rename("15_light" = ratio)
Si50_A_27_light2<- Si50_A_27_light[,c(1,2,4,5,7)]
Si50_A_27_light_CHH<-filter(Si50_A_27_light2, context=="CHH" & C_count>7)
Si50_A27<-Si50_A_27_light_CHH[,c(1,2,4)] %>%
  rename("27_light" = ratio)
Si50_A_35_light2<- Si50_A_35_light[,c(1,2,4,5,7)]
Si50_A_35_light_CHH<-filter(Si50_A_35_light2, context=="CHH" & C_count>7)
Si50_A35<-Si50_A_35_light_CHH[,c(1,2,4)] %>%
  rename("35_light" = ratio)
Si50_A_71_light2<- Si50_A_71_light[,c(1,2,4,5,7)]
Si50_A_71_light_CHH<-filter(Si50_A_71_light2, context=="CHH" & C_count>7)
Si50_A71<-Si50_A_71_light_CHH[,c(1,2,4)] %>%
  rename("71_light" = ratio)
Si50_A_89_light2<- Si50_A_89_light[,c(1,2,4,5,7)]
Si50_A_89_light_CHH<-filter(Si50_A_89_light2, context=="CHH" & C_count>7)
Si50_A89<-Si50_A_89_light_CHH[,c(1,2,4)] %>%
  rename("89_light" = ratio)
```

```
#Shade:

Si50_A_3_shade2<- Si50_A_3_shade[,c(1,2,4,5,7)]

Si50_A_3_shade_CHH<-filter(Si50_A_3_shade2, context=="CHH" & C_count>7)

Si50_A3<-Si50_A_3_shade_CHH[,c(1,2,4)] %>%

  rename("3_shade" = ratio)

Si50_A_41_shade2<- Si50_A_41_shade[,c(1,2,4,5,7)]

Si50_A_41_shade_CHH<-filter(Si50_A_41_shade2, context=="CHH" & C_count>7)

Si50_A41<-Si50_A_41_shade_CHH[,c(1,2,4)] %>%

  rename("41_shade" = ratio)

Si50_A_45_shade2<- Si50_A_45_shade[,c(1,2,4,5,7)]

Si50_A_45_shade_CHH<-filter(Si50_A_45_shade2, context=="CHH" & C_count>7)

Si50_A45<-Si50_A_45_shade_CHH[,c(1,2,4)] %>%

  rename("45_shade" = ratio)

Si50_A_47_shade2<- Si50_A_47_shade[,c(1,2,4,5,7)]

Si50_A_47_shade_CHH<-filter(Si50_A_47_shade2, context=="CHH" & C_count>7)

Si50_A47<-Si50_A_47_shade_CHH[,c(1,2,4)] %>%

  rename("47_shade" = ratio)

Si50_A_49_shade2<- Si50_A_49_shade[,c(1,2,4,5,7)]

Si50_A_49_shade_CHH<-filter(Si50_A_49_shade2, context=="CHH" & C_count>7)

Si50_A49<-Si50_A_49_shade_CHH[,c(1,2,4)] %>%

  rename("49_shade" = ratio)

Si50_A_105_shade2<- Si50_A_105_shade[,c(1,2,4,5,7)]

Si50_A_105_shade_CHH<-filter(Si50_A_105_shade2, context=="CHH" & C_count>7)

Si50_A105<-Si50_A_105_shade_CHH[,c(1,2,4)] %>%

  rename("105_shade" = ratio)


#Function to merge by "pos"= genomic location column


library(dplyr)

combine1<-merge(Ji98_L124, Ji98_L66, by="pos")

combine2<-merge(combine1, Ji98_L132, by="pos")
```

```
combine3<-merge(combine2, Ji98_L38, by="pos")
combine4<-merge(combine3, Ji98_L56, by="pos")
combine5<-merge(combine4, Ji98_L92, by="pos")
combine6<-merge(combine5, Ji98_L44, by="pos")
combine7<-merge(combine6, Ji98_L74, by="pos")
combine8<-merge(combine7, Ji98_L114,by="pos")
combine9<-merge(combine8, Ji98_L100,by="pos")
combine10<-merge(combine9, Ji98_L108, by="pos")
combine11<-merge(combine10, Ji98_L52, by="pos")

combine1<-merge(Ji98_A113, Ji98_A43, by="pos")
combine2<-merge(combine1, Ji98_A91, by="pos")
combine3<-merge(combine2, Ji98_A123, by="pos")
combine4<-merge(combine3, Ji98_A13, by="pos")
combine5<-merge(combine4, Ji98_A65, by="pos")
combine6<-merge(combine5, Ji98_A99, by="pos")
combine7<-merge(combine6, Ji98_A55, by="pos")
combine8<-merge(combine7, Ji98_A131,by="pos")
combine9<-merge(combine8, Ji98_A73,by="pos")
combine10<-merge(combine9, Ji98_A51, by="pos")
combine11<-merge(combine10, Ji98_A107, by="pos")

combine1<-merge(Ji75_L60, Ji75_L64, by="pos")#1,2
combine2<-merge(combine1, Ji75_L136, by="pos")#3
combine3<-merge(combine2, Ji75_L80, by="pos")#4
combine4<-merge(combine3, Ji75_L34, by="pos")#5
combine5<-merge(combine4, Ji75_L122, by="pos")#6
combine6<-merge(combine5, Ji75_L84, by="pos")#7
combine7<-merge(combine6, Ji75_L120, by="pos")#8
combine8<-merge(combine7, Ji75_L76,by="pos")#9
combine9<-merge(combine8, Ji75_L112,by="pos")#10
```

```
combine10<-merge(combine9, Ji75_L102, by="pos")#11
combine11<-merge(combine10, Ji75_L86, by="pos")#12

combine1<-merge(Ji75_A111, Ji75_A79, by="pos")#1,2
combine2<-merge(combine1, Ji75_A33, by="pos")#3
combine3<-merge(combine2, Ji75_A121, by="pos")#4
combine4<-merge(combine3, Ji75_A75, by="pos")#5
combine5<-merge(combine4, Ji75_A63, by="pos")#6
combine6<-merge(combine5, Ji75_A85, by="pos")#7
combine7<-merge(combine6, Ji75_A119, by="pos")#8
combine8<-merge(combine7, Ji75_A83,by="pos")#9
combine9<-merge(combine8, Ji75_A101,by="pos")#10
combine10<-merge(combine9, Ji75_A135, by="pos")#11
combine11<-merge(combine10, Ji75_A59, by="pos")#12

combine1<-merge(Si50_A89, Si50_A49, by="pos")#1,2
combine2<-merge(combine1, Si50_A15, by="pos")#3
combine3<-merge(combine2, Si50_A35, by="pos")#4
combine4<-merge(combine3, Si50_A45, by="pos")#5
combine5<-merge(combine4, Si50_A41, by="pos")#6
combine6<-merge(combine5, Si50_A9, by="pos")#7
combine7<-merge(combine6, Si50_A27, by="pos")#8
combine8<-merge(combine7, Si50_A3,by="pos")#9
combine9<-merge(combine8, Si50_A71,by="pos")#10
combine10<-merge(combine9, Si50_A47, by="pos")#11
combine11<-merge(combine10, Si50_A105, by="pos")#12

combine1<-merge(L165E_L126, L165E_L116, by="pos")#1,2
combine2<-merge(combine1, L165E_L128, by="pos")#3
combine3<-merge(combine2, L165E_L40, by="pos")#4
combine4<-merge(combine3, L165E_L2, by="pos")#5
```

```
combine5<-merge(combine4, L165E_L62, by="pos")#6

combine6<-merge(combine5, L165E_L8, by="pos")#7

combine7<-merge(combine6, L165E_L54, by="pos")#8

combine8<-merge(combine7, L165E_L78, by="pos")#9

combine9<-merge(combine8, L165E_L26, by="pos")#10

combine10<-merge(combine9, L165E_L110, by="pos")#11

combine11<-merge(combine10, L165E_L68, by="pos")#12


combine1<-merge(L165E_A115, L165E_A125, by="pos")#1,2

combine2<-merge(combine1, L165E_A61, by="pos")#3

combine3<-merge(combine2, L165E_A1, by="pos")#4

combine4<-merge(combine3, L165E_A127, by="pos")#5

combine5<-merge(combine4, L165E_A39, by="pos")#6

combine6<-merge(combine5, L165E_A7, by="pos")#7

combine7<-merge(combine6, L165E_A25, by="pos")#8

combine8<-merge(combine7, L165E_A53, by="pos")#9

combine9<-merge(combine8, L165E_A77, by="pos")#10

combine10<-merge(combine9, L165E_A67, by="pos")#11

combine11<-merge(combine10, L165E_A109, by="pos")#12


#Save the filtered cytosines by context in format .csv:


library(MASS)

write.matrix(Ji98_L_light, file = "Ji98_Leaves_CHH.csv", sep = ";")

write.matrix(combine11, file = "Ji98_Leaves_CHH_allsamples.csv", sep = ";")

write.matrix(combine11, file = "Ji98_Leaves_CHG_allsamples.csv", sep = ";")

write.matrix(combine11, file = "Ji98_Apex_CHH_context.csv", sep = ";")

write.matrix(combine11, file = "Ji98_Apex_CHG_context.csv", sep = ";")

write.matrix(combine11, file = "Si50_Apex_CHG_context.csv", sep = ";")

write.matrix(combine11, file = "Si50_Apex_CHH_context.csv", sep = ";")

write.matrix(combine11, file = "165E_Leaves_CHH_context.csv", sep = ";")
```

```
write.matrix(combine11, file = "165E_Leaves_CHG_context.csv", sep = ";")
write.matrix(combine11, file = "165E_Apex_CHG_context.csv", sep = ";")
write.matrix(combine11, file = "165E_Apex_CHH_context.csv", sep = ";")

write.matrix(combine11, file = "Ji75_Leaves_CHH_context.csv", sep = ";")#do PCA
write.matrix(combine11, file = "Ji75_Leaves_CHG_context.csv", sep = ";")#do PCA
write.matrix(combine11, file = "Ji75_Apex_CHH_context.csv", sep = ";")#do PCA
write.matrix(combine11, file = "Ji75_Apex_CHG_context.csv", sep = ";")#do PCA
```

```
#Read csv files per CHH or CHG context for each line and condition:

library(readr)
Ji75_Leaves_CHH<-read_delim("Ji75_Leaves_CHH_context.csv", ";", escape_double = FALSE, trim_ws = TRUE)
Ji75_Leaves_CHG<-read_delim("Ji75_Leaves_CHG_context.csv", ";", escape_double = FALSE, trim_ws = TRUE)
Ji75_Apex_CHH<-read_delim("Ji75_Apex_CHH_context.csv", ";", escape_double = FALSE, trim_ws = TRUE)
Ji75_Apex_CHG<-read_delim("Ji75_Apex_CHG_context.csv", ";", escape_double = FALSE, trim_ws = TRUE)
Ji98_Leaves_CHH <- read_delim("Ji98_Leaves_CHH_allsamples.csv", ";", escape_double = FALSE, trim_ws = TRUE)
Ji98_Leaves_CHG <- read_delim("Ji98_Leaves_CHG_allsamples.csv", ";", escape_double = FALSE, trim_ws = TRUE)
Ji98_Apex_CHH <- read_delim("Ji98_Apex_CHH_context.csv", ";", escape_double = FALSE, trim_ws = TRUE)
Ji98_Apex_CHG <- read_delim("Ji98_Apex_CHG_context.csv", ";", escape_double = FALSE, trim_ws = TRUE)
Si50_Apex_CHG <- read_delim("Si50_Apex_CHG_context.csv", ";", escape_double = FALSE, trim_ws = TRUE)
Si50_Apex_CHH <- read_delim("Si50_Apex_CHH_context.csv", ";", escape_double = FALSE, trim_ws = TRUE)
L165E_Leaves_CHH <- read_delim("165E_Leaves_CHH_context.csv", ";", escape_double = FALSE, trim_ws = TRUE)
```

```
L165E_Leaves_CHG <- read_delim("165E_Leaves_CHG_context.csv", ";", escape_double = FALSE, trim_ws = TRUE)

L165E_Apex_CHG <- read_delim("165E_Apex_CHG_context.csv", ";", escape_double = FALSE, trim_ws = TRUE)

L165E_Apex_CHH <- read_delim("165E_Apex_CHH_context.csv", ";", escape_double = FALSE, trim_ws = TRUE)

Ji75_Leaves_CHH2<-Ji75_Leaves_CHH[,c(3,5,7,9,11,13,15,17,19,21,23,25)]

Ji75_Leaves_CHG2<-Ji75_Leaves_CHG[,c(3,5,7,9,11,13,15,17,19,21,23,25)]

Ji75_Apex_CHH2<-Ji75_Apex_CHH[,c(3,5,7,9,11,13,15,17,19,21,23,25)]

Ji75_Apex_CHG2<-Ji75_Apex_CHG[,c(3,5,7,9,11,13,15,17,19,21,23,25)]

Ji98_Leaves_CHH2<-Ji98_Leaves_CHH[,c(3,5,7,9,11,13,15,17,19,21,23,25)]

Ji98_Leaves_CHG2<-Ji98_Leaves_CHG[,c(3,5,7,9,11,13,15,17,19,21,23,25)]

Ji98_Apex_CHH2<-Ji98_Apex_CHH[,c(3,5,7,9,11,13,15,17,19,21,23,25)]

Ji98_Apex_CHG2<-Ji98_Apex_CHG[,c(3,5,7,9,11,13,15,17,19,21,23,25)]

Si50_Apex_CHG2<-Si50_Apex_CHG[,c(3,5,7,9,11,13,15,17,19,21,23,25)]

Si50_Apex_CHH2<-Si50_Apex_CHH[,c(3,5,7,9,11,13,15,17,19,21,23,25)]

L165E_Leaves_CHH2<-L165E_Leaves_CHH[,c(3,5,7,9,11,13,15,17,19,21,23,25)]

L165E_Leaves_CHG2<-L165E_Leaves_CHG[,c(3,5,7,9,11,13,15,17,19,21,23,25)]

L165E_Apex_CHG2<-L165E_Apex_CHG[,c(3,5,7,9,11,13,15,17,19,21,23,25)]

L165E_Apex_CHH2<-L165E_Apex_CHH[,c(3,5,7,9,11,13,15,17,19,21,23,25)]


#Applied PCA analysis


library("FactoMineR")

library("factoextra")

library(emmeans)

library(missMDA)


imp.Ji75_L<-imputePCA(Ji75_Leaves_CHH2, ncp=10)

imp.Ji75_L<-imputePCA(Ji75_Leaves_CHG2, ncp=10)

imp.Ji75_A<-imputePCA(Ji75_Apex_CHH2, ncp=10)

imp.Ji75_A<-imputePCA(Ji75_Apex_CHG2, ncp=10)
```

```
imp.Ji98_L<-imputePCA(Ji98_Leaves_CHG2, ncp=10)

imp.Ji98_A<-imputePCA(Ji98_Apex_CHH2, ncp=10)

imp.Ji98_Achg<-imputePCA(Ji98_Apex_CHG2, ncp=10)

imp.Si50_A<-imputePCA(Si50_Apex_CHG2, ncp=10)

imp.165E_L<-imputePCA(L165E_Leaves_CHG2, ncp=10)

imp.165E_A<-imputePCA(L165E_Apex_CHH2, ncp=10)

imp.165E_A<-imputePCA(L165E_Apex_CHG2, ncp=10)


###### extract imputed observations

Ji98L.CHH<-imp.Ji98_L$completeObs

Ji98A.CHH<-imp.Ji98_A$completeObs

Ji98A.CHG<-imp.Ji98_Achg$completeObs

Ji98L.CHG<-imp.Ji98_L$completeObs

Si50A.CHG<-imp.Si50_A$completeObs

Si50A.CHH<-imp.Si50_A$completeObs

L165EL.CHG<-imp.165E_L$completeObs

L165EA.CHG<-imp.165E_A$completeObs

Ji75L.CHH<-imp.Ji75_L$completeObs

Ji75L.CHG<-imp.Ji75_L$completeObs

Ji75A.CHH<-imp.Ji75_A$completeObs

Ji75A.CHG<-imp.Ji75_A$completeObs


###### transpose the data. We need samples in rows and C's in columns


Ji98L.CHG<-as.data.frame(t(as.matrix(imp.Ji98_L)))

Ji98A.CHG<-as.data.frame(t(as.matrix(imp.Ji98_Achg)))

Si50A.CHG<-as.data.frame(t(as.matrix(imp.Si50_A)))

Si50A.CHG<-as.data.frame(t(as.matrix(imp.Si50_A)))

L165EL.CHH<-as.data.frame(t(as.matrix(imp.165E_L)))

L165EL.CHG<-as.data.frame(t(as.matrix(imp.165E_L)))

L165EA.CHG<-as.data.frame(t(as.matrix(imp.165E_A)))
```

```
L165EL.CHG<-as.data.frame(t(as.matrix(imp.165E_L)))

Ji75L.CHH<- as.data.frame(t(as.matrix(Ji75_Leaves_CHH2)))

Ji75L.CHG<- as.data.frame(t(as.matrix(imp.Ji75_L)))

Ji75A.CHH<- as.data.frame(t(as.matrix(imp.Ji75_A)))

Ji75A.CHG<- as.data.frame(t(as.matrix(imp.Ji75_A)))


#Change rownames:


rownames(Ji98L.CHH)<- c("124_shade", "66_light", "132_light",  "38_light",  "56_light", "92_shade","44_shade", "74_light",

                    "114_light", "100_shade", "108_shade", "52_light")

rownames(Ji98L.CHG)<- c("124_shade", "66_light",  "38_light", "132_light", "56_light", "92_shade", "74_light", "44_shade",

                    "114_light", "100_shade", "108_shade", "52_light")

rownames(Ji98A.CHH)<- c("113_light", "123_shade", "99_shade", "91_shade", "43_shade", "65_light",

                    "55_light", "13_light","131_light", "73_light", "51_shade", "107_shade")

rownames(Ji98A.CHG)<- c("113_light", "43_shade", "91_shade", "123_shade", "13_light", "65_light", "99_shade",

                    "55_light", "131_light", "73_light", "51_shade", "107_shade")

rownames(Si50A.CHH)<- c("49_shade", "89_light", "15_light","45_shade","35_light","9_light","27_light", "3_shade", "71_light", "47_shade",

                    "105_shade")

rownames(L165E.CHH)<- c("126_shade", "128_shade", "116_shade","40_light","2_light","8_light","78_shade", "62_shade", "54_light", "26_light", "110_shade",

                    "68_shade")


# Do PCA


library(FactoMineR)


pca.Ji98L <- PCA(Ji98L.CHG, scale.unit=TRUE, ncp=11, graph=FALSE)

pca.Ji98A <- PCA(Ji98A.CHG, scale.unit=TRUE, ncp=11, graph=FALSE)
```

```
pca.Si50A <- PCA(Si50A.CHG, scale.unit=TRUE, ncp=11, graph=FALSE)
pca.165EL <- PCA(L165EL.CHG, scale.unit=TRUE, ncp=11, graph=FALSE)
pca.165EA <- PCA(L165EA.CHG, scale.unit=TRUE, ncp=11, graph=FALSE)
pca.Ji75L <- PCA(Ji75L.CHH, scale.unit=TRUE, ncp=11, graph=FALSE)
pca.Ji75L <- PCA(Ji75L.CHG, scale.unit=TRUE, ncp=11, graph=FALSE)
pca.Ji75A <- PCA(Ji75A.CHH, scale.unit=TRUE, ncp=11, graph=FALSE)
pca.Ji75A <- PCA(Ji75A.CHG, scale.unit=TRUE, ncp=11, graph=FALSE)

#Plot PCAs
y.CHG.80<-rownames(L165E.CHG)
label.CHG.80<-do.call(rbind, strsplit(y.CHG.80, '_'))
L165E.CHG$Treatment<-label.CHG.80[,2]
plot.L165E<-fviz_pca_ind(pca.165EL, geom.ind =c("point"),
                 col.ind=L165E.CHG$Treatment, repel=TRUE, title="165E Apex CHG context")
print(plot.L165E)

y.CHH.80<-rownames(L165Ea.CHH)
label.CHH.80<-do.call(rbind, strsplit(y.CHH.80, '_'))
L165Ea.CHH$Treatment<-label.CHH.80[,2]
plot.L165E<-fviz_pca_ind(pca.165EA, geom.ind =c("point"),
                col.ind=L165Ea.CHH$Treatment, repel=TRUE, title="165E Apex CHH context")
print(plot.L165E)


y.CHH.80<-rownames(Si50A.CHH)
label.CHH.80<-do.call(rbind, strsplit(y.CH.80, '_'))
Si50A.CHH$Treatment<-label.CHG.80[,2]
plot.Si50A<-fviz_pca_ind(pca.Si50A, geom.ind =c("point"),
                col.ind=Si50A.CHG$Treatment, repel=TRUE, title="Si50 Apex CHH context")
print(plot.Si50A)
```

```
y.CHH.80<-rownames(Ji98A.CHH)

label.CHH.80<-do.call(rbind, strsplit(y.CHH.80, '_'))

Ji98A.CHH$Treatment<-label.CHH.80[,2]

plot.Ji98A<-fviz_pca_ind(pca.Ji98A, geom.ind =c("point"),

                       col.ind=Ji98A.CHH$Treatment, repel=TRUE, title="Line Ji98 Apex CHH context")

print(plot.Ji98A)

y.CHG.80<-rownames(Ji75L.CHG)

label.CHG.80<-do.call(rbind, strsplit(y.CHG.80, '_'))

Ji75L.CHG$Treatment<-label.CHG.80[,2]

plot.Ji75L<-fviz_pca_ind(pca.Ji75L, geom.ind =c("point"),

                       col.ind=Ji75L.CHG$Treatment, repel=TRUE, title="Ji75 Leaves CHG context")

y.CHH.80<-rownames(Ji75A.CHH)

label.CHH.80<-do.call(rbind, strsplit(y.CHH.80, '_'))

Ji75A.CHH$Treatment<-label.CHH.80[,2]

plot.Ji75A<-fviz_pca_ind(pca.Ji75A, geom.ind =c("point"),

                       col.ind=Ji75A.CHH$Treatment, repel=TRUE, title="Ji75 Apex CHH context")

#Save with labels


plot.Ji98L2<-fviz_pca_ind(pca.Ji98L, geom.ind =c("point", "text"),

                        col.ind=Ji98L.CHG$Treatment, repel=TRUE, title="Line Ji98 Tissue Leaves -CHH
methylation context")

print(plot.Ji98L2)

print(pca.Ji98L)

print(pca.Ji98A)

print(pca.165EL)

print(pca.165EA)

print(pca.Ji75L)

print(pca.Ji75A)


# Obtain Eigenvalues and contribution to variance by component
```

```
library(factoextra)

eig.val<-get_eigenvalue(pca.165EA)

eig.val

fviz_eig(pca.Ji98A, addlabels = TRUE, ylim=c(0,50))

var<-get_pca_var(pca.165EA)

print(var)

head(var$coord, 4)

fviz_pca_var(pca.Ji98A, col.var = "black")

library(corrplot)

corrplot(var$cos2, is.corr = FALSE)

ind<-get_pca_ind(pca.165EA)

print(ind)

ind

head(ind$coord)

head(ind$cos2)

fviz_pca_ind(pca.Ji98L)


#To plot PCA

fviz_pca_ind(pca.Ji75A,

        geom.ind = "point",

        title="Line Ji75 Tissue Apex -CHH methylation context",

        col.ind = Ji75A.CHH$Treatment,

        palette = c("#000000","#0072B2"),

        addEllipses = TRUE,

        legend.title = "Treatment")


#To save PCA data in a matrix

library(MASS)

coordJi98_L_CHH<-as.matrix(ind[["coord"]])

coordJi98_L_CHG<-as.matrix(ind[["coord"]])

coordJi98_A_CHH<-as.matrix(ind[["coord"]])
```

```
coordJi98_A_CHG<-as.matrix(ind[["coord"]])
coordSi50_A_CHG<-as.matrix(ind[["coord"]])
coordSi50_A_CHH<-as.matrix(ind[["coord"]])
coord165E_L_CHH<-as.matrix(ind[["coord"]])
coord165E_L_CHG<-as.matrix(ind[["coord"]])
coord165E_A_CHG<-as.matrix(ind[["coord"]])
coord165E_A_CHH<-as.matrix(ind[["coord"]])
coordJi75_L_CHH<- as.matrix(ind[["coord"]])
coordJi75_L_CHG<- as.matrix(ind[["coord"]])
coordJi75_A_CHG<- as.matrix(ind[["coord"]])
coordJi75_A_CHH<- as.matrix(ind[["coord"]])
contribJi98_A_CHH<-as.matrix(var[["contrib"]])
contribJi98_A_CHG<-as.matrix(var[["contrib"]])
contribJi98_L_CHG<-as.matrix(var[["contrib"]])
contribSi50_A_CHG<-as.matrix(var[["contrib"]])
contribSi50_A_CHH<-as.matrix(var[["contrib"]])
contrib165E_L_CHH<-as.matrix(var[["contrib"]])
contrib165E_L_CHG<-as.matrix(var[["contrib"]])
contrib165E_A_CHG<-as.matrix(var[["contrib"]])
contrib165E_A_CHH<-as.matrix(var[["contrib"]])
contribJi75_L_CHH<-as.matrix(var[["contrib"]])
contribJi75_L_CHG<-as.matrix(var[["contrib"]])
contribJi75_A_CHH<-as.matrix(var[["contrib"]])
contribJi75_A_CHG<-as.matrix(var[["contrib"]])
write.matrix(coordJi98_L_CHH, file = "Coordonnees_ACP_Ji98_Leaves_CHH.csv", sep = ";")
write.matrix(coordJi98_L_CHG, file = "Coordonnees_ACP_Ji98_Leaves_CHG.csv", sep = ";")
write.matrix(coordJi98_A_CHH, file = "Coordonnees_ACP_Ji98_Apex_CHH.csv", sep = ";")
write.matrix(coordJi98_A_CHG, file = "Coordonnees_ACP_Ji98_Apex_CHG.csv", sep = ";")
write.matrix(coordSi50_A_CHG, file = "Coordonnees_ACP_Si50_Apex_CHG.csv", sep = ";")
write.matrix(coordSi50_A_CHH, file = "Coordonnees_ACP_Si50_Apex_CHH.csv", sep = ";")
write.matrix(coord165E_L_CHH, file = "Coordonnees_ACP_165E_Leaves_CHH.csv", sep = ";")
```

```
write.matrix(coord165E_L_CHG, file = "Coordonnees_ACP_165E_Leaves_CHG.csv", sep = ";")

write.matrix(coord165E_A_CHG, file = "Coordonnees_ACP_165E_Apex_CHG.csv", sep = ";")

write.matrix(coord165E_A_CHH, file = "Coordonnees_ACP_165E_Apex_CHH.csv", sep = ";")

write.matrix(coordJi75_L_CHH, file = "Coordonnees_ACP_Ji75_Leaves_CHH.csv", sep = ";")

write.matrix(coordJi75_L_CHG, file = "Coordonnees_ACP_Ji75_Leaves_CHG.csv", sep = ";")

write.matrix(coordJi75_A_CHH, file = "Coordonnees_ACP_Ji75_Apex_CHH.csv", sep = ";")

write.matrix(coordJi75_A_CHG, file = "Coordonnees_ACP_Ji75_Apex_CHG.csv", sep = ";")

write.matrix(contribJi98_A_CHH, file = "Contribution_des_variables_aux_axes_Ji98_Apex_CHH.csv",
sep = ";")

write.matrix(contribJi98_A_CHG, file = "Contribution_des_variables_aux_axes_Ji98_Apex_CHG.csv",
sep = ";")

write.matrix(contribJi98_L_CHG, file = "Contribution_des_variables_aux_axes_Ji98_Leaves_CHG.csv",
sep = ";")

write.matrix(contribSi50_A_CHG, file = "Contribution_des_variables_aux_axes_Si50_Apex_CHG.csv",
sep = ";")

write.matrix(contribSi50_A_CHH, file = "Contribution_des_variables_aux_axes_Si50_Apex_CHH.csv",
sep = ";")

write.matrix(contrib165E_L_CHG, file =
"Contribution_des_variables_aux_axes_165E_Leaves_CHG.csv", sep = ";")

write.matrix(contrib165E_A_CHG, file = "Contribution_des_variables_aux_axes_165E_Apex_CHG.csv",
sep = ";")

write.matrix(contrib165E_A_CHH, file = "Contribution_des_variables_aux_axes_165E_Apex_CHH.csv",
sep = ";")

write.matrix(contribJi75_L_CHH, file = "Contribution_des_variables_aux_axes_Ji75_Leaves_CHH.csv",
sep = ";")

write.matrix(contribJi75_L_CHG, file = "Contribution_des_variables_aux_axes_Ji75_Leaves_CHG.csv",
sep = ";")

write.matrix(contribJi75_A_CHH, file = "Contribution_des_variables_aux_axes_Ji75_Apex_CHH.csv",
sep = ";")

write.matrix(contribJi75_A_CHG, file = "Contribution_des_variables_aux_axes_Ji75_Apex_CHG.csv",
sep = ";")

contrib165E_L_CHG<-as.matrix(var[["contrib"]])

eig.valJi98A.CHH<-eig.val

eig.valJi98A.CHG<-eig.val

write.matrix(eig.valJi98CHH, file = "Contributions_par_composant_Ji98_Leaves_CHH.csv", sep = ";")
```

```
write.matrix(eig.val, file = "Contributions_par_composant_Ji98_Leaves_CHG.csv", sep = ";")
write.matrix(eig.valJi98A.CHH, file = "Contributions_par_composant_Ji98_Apex_CHH.csv", sep = ";")
write.matrix(eig.valJi98A.CHG, file = "Contributions_par_composant_Ji98_Apex_CHG.csv", sep = ";")
write.matrix(eig.val, file = "Contributions_par_composant_Si50_Apex_CHG.csv", sep = ";")
write.matrix(eig.val, file = "Contributions_par_composant_Si50_Apex_CHH.csv", sep = ";")
write.matrix(eig.val, file = "Contributions_par_composant_165E_Leaves_CHH.csv", sep = ";")
write.matrix(eig.val, file = "Contributions_par_composant_165E_Leaves_CHG.csv", sep = ";")
write.matrix(eig.val, file = "Contributions_par_composant_165E_Apex_CHG.csv", sep = ";")
write.matrix(eig.val, file = "Contributions_par_composant_165E_Apex_CHH.csv", sep = ";")
write.matrix(eig.val, file = "Contributions_par_composant_Ji75_Leaves_CHH.csv", sep = ";")
write.matrix(eig.val, file = "Contributions_par_composant_Ji75_Leaves_CHG.csv", sep = ";")
write.matrix(eig.val, file = "Contributions_par_composant_Ji75_Apex_CHG.csv", sep = ";")
write.matrix(eig.val, file = "Contributions_par_composant_Ji75_Apex_CHH.csv", sep = ";")
```

```
#################################################
###
###     Data Epigenetics Antirrhinum majus
###       Data Analysis
###         - 1 Phenotypic traits -
###
#################################################


rm(list = ls())


library(PerformanceAnalytics)
library(viridis)
library(scales)
library(rcompanion)
library(coin)


##  Load Pheno Data
Pheno <- read.csv("ENSFEA2 DATA working Pierick.csv", sep = ";")


str(Pheno)





###----------------
###
### differences due to treatment
### for each line, each trait
###
```

```
###----------------

###
### Function that calculates effect sizes
###  for each line, each trait
###
func.test.effsize.treat.Pheno <- function(Dataset.func, N.boot){

 line <- expand.grid(levels(Dataset.func$Line), colnames(Dataset.func[, 8 : ncol(Dataset.func)]))[, 1]
 trait <- expand.grid(levels(Dataset.func$Line), colnames(Dataset.func[, 8 : ncol(Dataset.func)]))[, 2]

 ResTab.func <- matrix(nrow = length(line), ncol = 5)
 dimnames(ResTab.func)[[2]] <- c("line", "trait", "effsize", "conf.low", "conf.high")
 ResTab.func <- data.frame(ResTab.func)
 ResTab.func[, "line"] <- as.character(line)
 ResTab.func[, "trait"] <- as.character(trait)



 subtab <- Dataset.func[, c("Name", "Treatment",  "Line")]



 for(i in 1 : length(ResTab.func$line)){

  trait.Temp.tab <- data.frame(subtab[subtab$Line == ResTab.func$line[i], ],
                               Dataset.func[Dataset.func$Line == ResTab.func$line[i], paste(ResTab.func$trait[i])])
  dimnames(trait.Temp.tab)[[2]][4] <- "Trait.x"

  temp <- rep(NA, 3)

  while(is.na(temp[2]) == TRUE){
   temp <- wilcoxonR(x = trait.Temp.tab$Trait.x, g = trait.Temp.tab$Treatment, ci = TRUE, R = N.boot)
```

```r
      ResTab.func[i, 3 : 5] <- as.numeric(temp)

    }
  }
  return(ResTab.func)
}


###
### Calculate effect sizes
###

start <- Sys.time()
tab_effsize_Pheno <- func.test.effsize.treat.Pheno(Pheno, N.boot = 5000)
end <- Sys.time()
end - start
write.table(x = tab_effsize_Pheno, file = "tab_effsize_Pheno.txt")



###
###  GRAPH function
###  for each line, each treatment
###

## load data table "tab_effsize_Pheno.txt"
tab_effsize_Pheno <- read.table(file = "tab_effsize_Pheno.txt")

levels(tab_effsize_Pheno$trait)[3] <- "Mean internode length"
levels(tab_effsize_Pheno$trait)[4] <- "Number of branches"
levels(tab_effsize_Pheno$trait)[6] <- "Total leaves number"
```

```r
colnames(Pheno)[10] <- "Number\nof\nbranches"
colnames(Pheno)[11] <- "Mean\ninternode\nlength"
colnames(Pheno)[13] <- "Total\nleaves\nnumber"



### ---------------
### FIGURE 1
### ---------------

### Function that makes the plot
func.graph.effsize.Pheno.Bytrait.VERT <- function(Datatab.Graph, ShowLegend){
  par(mar = c(5, 9, 2, 2), xpd = FALSE, cex = 1.5)

  # nb of lines
  nb.lines <- length(levels(as.factor(Datatab.Graph$line)))

  temp.vect.incl <- func.vect.ci.incl(Datatab.Graph)

  ## y.axis position
  y.axis.pos <- seq(1, length.out = nrow(Datatab.Graph), by = 0.5)


  plot(y.axis.pos ~ Datatab.Graph$effsize, xlim = c(-1, 1.15), type = "n", axes = F, xlab = "", ylab = "")

  abline(v = 0, lwd = 2)
  abline(v = c(-0.3, -0.5, 0.3, 0.5), lty = 3, col = "gray50")

  polygon(y = c(y.axis.pos[5] - 0.25, y.axis.pos[5] - 0.25, y.axis.pos[8] + 0.25, y.axis.pos[8] + 0.25),
          x = c(-1, 1.15, 1.15, -1),
          col = alpha("gray", 0.3), border = NA)
```

```
polygon(y = c(y.axis.pos[13] - 0.25, y.axis.pos[13] - 0.25, y.axis.pos[16] + 0.25, y.axis.pos[16] + 0.25),
        x = c(-1, 1.15, 1.15, -1),
        col = alpha("gray", 0.3), border = NA)
polygon(y = c(y.axis.pos[21] - 0.25, y.axis.pos[21] - 0.25, y.axis.pos[24] + 0.25, y.axis.pos[24] + 0.25),
        x = c(-1, 1.15, 1.15, -1),
        col = alpha("gray", 0.3), border = NA)


segments(y0 = y.axis.pos, y1 = y.axis.pos,
         x0 = Datatab.Graph$conf.low, x1 = Datatab.Graph$conf.high,
         lwd = 2)
points(y.axis.pos ~ Datatab.Graph$effsize,
       pch = ifelse(temp.vect.incl == 0, 19, 1),
       cex = ifelse(temp.vect.incl == 0, 1.3, 1))




axis(side = 1, at = seq(-1, 1, 0.2), cex.axis = 1.5)
box()
mtext(text = colnames(Pheno)[10 : ncol(Pheno)],
      side = 2, line = 3.5, las = 2, adj = 0.5,
      at = seq(1.75, 11.75, length.out = 6),
      cex = 2)

mtext(text = "Effect size, r",
      side = 1, line = 3,
      cex = 2.5)

mtext(text = "Traits",
      side = 2, line = 7,
      cex = 2.5)
```

```
  if(ShowLegend == "yes"){

    text(labels = levels(as.factor(Datatab.Graph$line)),
         y = y.axis.pos, x = 1.05, cex = 1)

  }
}

### Plot FIGURE 1
png(filename = "PhenoEffSize_bytrait_VERT.png", width = 10, height = 12, units = 'in', res = 300)

func.graph.effsize.Pheno.Bytrait.VERT(tab_effsize_Pheno, "yes")

dev.off()

##################################################
###
###    - 2 Methylation ~ light treatment
###       PCA Coordinates
###
##################################################
```

```
##  Load PCA Data

Apex.CHG <- read.csv("Coordonnees_ACP_Compil_Apex_CHG.csv", sep = ";")

Apex.CHH <- read.csv("Coordonnees_ACP_Compil_Apex_CHH.csv", sep = ";")

Apex.CpG <- read.csv("Coordonnees_ACP_Compil_Apex_CpG.csv", sep = ";")



Leaves.CHG <- read.csv("Coordonnees_ACP_Compil_Leaves_CHG.csv", sep = ";")

Leaves.CHH <- read.csv("Coordonnees_ACP_Compil_Leaves_CHH.csv", sep = ";")

Leaves.CpG <- read.csv("Coordonnees_ACP_Compil_Leaves_CpG.csv", sep = ";")



str(Apex.CHG)

str(Apex.CHH)

str(Apex.CpG)


str(Leaves.CHG)

str(Leaves.CHH)

str(Leaves.CpG)




###----------------

###

### test differences between treatments for each Line

###   in each methyl protocol, and each tissu (Apex, Leaves)

###

###----------------


###
```

```
### Function: Extract effect size and CI, and fill output table
###
func.test.fill.restab <- function(Dataset.func, N.boot){


  line <- expand.grid(levels(Dataset.func$Line), colnames(Dataset.func[, 4 : ncol(Dataset.func)])))[, 1]
  dim <- expand.grid(levels(Dataset.func$Line), colnames(Dataset.func[, 4 : ncol(Dataset.func)])))[, 2]
  ResTab.func <- matrix(nrow = length(line), ncol = 5)
  dimnames(ResTab.func)[[2]] <- c("line", "Dim", "effsize", "conf.low", "conf.high")
  ResTab.func <- data.frame(ResTab.func)
  ResTab.func[, "line"] <- as.character(line)
  ResTab.func[, "Dim"] <- as.character(dim)


  subtab <- Dataset.func[, 1 : 3]
  # N.boot <- 1000


  for(i in 1: length(ResTab.func$line)){

    Dim.Temp.tab <- data.frame(subtab[subtab$Line == ResTab.func$line[i], ],
Dataset.func[Dataset.func$Line == ResTab.func$line[i], paste(ResTab.func$Dim[i])])
    dimnames(Dim.Temp.tab)[[2]][4] <- "Dim.x"

    temp <- rep(NA, 3)

    while(is.na(temp[2]) == TRUE){
      temp <- wilcoxonR(x = Dim.Temp.tab$Dim.x, g = Dim.Temp.tab$Treatment, ci = TRUE, R = N.boot)
      ResTab.func[i, 3 : 5] <- as.numeric(temp)

    }
```

```
  }
  return(ResTab.func)
}


###
### function checks if 0 is included in CI: (0: zero not included, 1 = zero included)
###
func.vect.ci.incl <- function(Data.tab){

  vect.ci.incl <- numeric(length(Data.tab$line))

  for(i in 1 : length(Data.tab$line)){
    # if effsize > 0, 0 not included if conf.low > 0
    # 1 means 0 included
    if(isTRUE(Data.tab$effsize[i] > 0) == T){

      vect.ci.incl[i] <- ifelse(test = Data.tab$conf.low[i] > 0, 0, 1)

    } else if(isTRUE(Data.tab$effsize[i] < 0) == T){    # if effsize < 0, 0 not included if conf.high < 0

      vect.ci.incl[i] <- ifelse(test = Data.tab$conf.high[i] < 0, 0, 1)

    } else if(isTRUE(Data.tab$effsize[i] == 0) == T){

      vect.ci.incl[i] <- 1

    }
  }
  return(vect.ci.incl)
}
```

```
###
### function that makes the plot
###

func.graph.effsize <- function(Datatab.Graph, ShowLegend, ShowYAxisLab){
  par(mar = c(5, 5, 3, 2), xpd = FALSE, cex = 1.5)

  # nb of lines
  nb.lines <- length(levels(as.factor(Datatab.Graph$line)))
  nb.dim <- length(levels(as.factor(Datatab.Graph$Dim)))

  # Sort table for plotting by lines on x-axis
  plot.title <- strsplit(paste(deparse(substitute(Datatab.Graph))), split = "_")[[1]][2]
  Datatab.Graph <- Datatab.Graph[order(Datatab.Graph$line), ]



  # vector CI includes 0: Y/N (1: zero included in CI)
  temp.vect.incl <- func.vect.ci.incl(Datatab.Graph)

  ## y.axis position
  # y.axis.pos <- seq(1, length.out = nrow(Datatab.Graph), by = 0.5)
  y.axis.pos <- 1 : nrow(Datatab.Graph)



  plot(y.axis.pos ~ Datatab.Graph$effsize, xlim = c(-1, 1.1), axes = F, type = "n",
       xlab = "Effect size, r", ylab = "", cex.lab = 1.5,
       main = paste(plot.title))

  abline(v = 0, lwd = 2)
  abline(v = 0.3, lty = 3, col = "gray50")
  abline(v = 0.5, lty = 3, col = "gray50")
```

```
abline(v = -0.3, lty = 3, col = "gray50")
abline(v = -0.5, lty = 3, col = "gray50")


polygon(y = c(0.5, 0.5, nb.dim + 0.5, nb.dim + 0.5),
        x = c(-1, 1.1, 1.1, -1),
        col = alpha("gray", 0.3), border = NA)


polygon(y = c((nb.dim * 2) + 0.5, (nb.dim * 2) + 0.5, (nb.dim * 3) + 0.5, (nb.dim * 3) + 0.5),
        x = c(-1, 1.1, 1.1, -1),
        col = alpha("gray", 0.3), border = NA)


segments(y0 = rev(y.axis.pos), y1 = rev(y.axis.pos),
         x0 = Datatab.Graph$conf.low, x1 = Datatab.Graph$conf.high,
         lwd = 2)

points(rev(y.axis.pos) ~ (Datatab.Graph$effsize),
       pch = ifelse(temp.vect.incl == 0, 19, 1), cex = ifelse(temp.vect.incl == 0, 1.7, 1.2))


axis(side = 1, at = seq(-1, 1, 0.2), cex = 2)
box()
mtext(text = rev(levels(as.factor(Datatab.Graph$line))),
      side = 2, line = 0.5, cex = 2, las = 2,
      at = c(seq(ifelse(nb.dim < 12, yes = 6, no = 6.5), by = nb.dim, length.out =
length(levels(as.factor(Datatab.Graph$line))))))


if(ShowLegend == "yes"){
```

```
    text(labels = rev(seq(1, nb.dim, 1)),
         y = y.axis.pos, x = 1, cex = 0.7)
  }

  if(ShowYAxisLab == "yes"){

    mtext(text = "Lines",
          side = 2, line = 3.5, cex = 2.5)

  }
}


###----------------
###
###   Calcultate Effect sizes, output tables
###
###----------------

### Apex
start <- Sys.time()
tab_ApexCHG <- func.test.fill.restab(Apex.CHG, N.boot = 5000)
# write.table(x = tab_ApexCHG, file = "tab_ApexCHG.txt")
end <- Sys.time()
end - start

start <- Sys.time()
tab_ApexCHH <- func.test.fill.restab(Apex.CHH, N.boot = 5000)
# write.table(x = tab_ApexCHH, file = "tab_ApexCHH.txt")
end <- Sys.time()
```

```
end - start

start <- Sys.time()
tab_ApexCpG <- func.test.fill.restab(Apex.CpG, N.boot = 5000)
# write.table(x = tab_ApexCpG, file = "tab_ApexCpG.txt")
end <- Sys.time()
end - start



### Leaves
start <- Sys.time()
tab_LeaveCHG <- func.test.fill.restab(Leaves.CHG, N.boot = 5000)
end <- Sys.time()
end - start
# write.table(x = tab_LeaveCHG, file = "tab_LeaveCHG.txt")

start <- Sys.time()
tab_LeaveCHH <- func.test.fill.restab(Leaves.CHH, N.boot = 5000)
end <- Sys.time()
end - start
# write.table(x = tab_LeaveCHH, file = "tab_LeaveCHH.txt")

start <- Sys.time()
tab_LeaveCpG <- func.test.fill.restab(Leaves.CpG, N.boot = 5000)
end <- Sys.time()
end - start
# write.table(x = tab_LeaveCpG, file = "tab_LeaveCpG.txt")
```

```
###----------------
###
###   GRPAHS , output table
###
###----------------

# Load tables
tab_ApexCHG <- read.table(file = "tab_ApexCHG.txt")
tab_ApexCHH <- read.table(file = "tab_ApexCHH.txt")
tab_ApexCpG <- read.table(file = "tab_ApexCpG.txt")

tab_LeaveCHG <- read.table(file = "tab_LeaveCHG.txt")
tab_LeaveCHH <- read.table(file = "tab_LeaveCHH.txt")
tab_LeaveCpG <- read.table(file = "tab_LeaveCpG.txt")



### ---------------------------------------
###
###   FIGURE 2: Apex
###
### ---------------------------------------

png(filename = "Apex_3plot_byLine.png", width = 18, height = 18, units = 'in', res = 300)

par(mfrow = c(1, 3))
func.graph.effsize(tab_ApexCHG, "yes", "yes")
```

```
func.graph.effsize(tab_ApexCHH, "yes", "no")
func.graph.effsize(tab_ApexCpG, "yes", "no")

dev.off()




### ---------------------------------------
###
###   FIGURE 3: Leaves
###
### ---------------------------------------

png(filename = "Leaves_3plot_byLine.png", width = 16, height = 16, units = 'in', res = 300)

par(mfrow = c(1, 3))
func.graph.effsize(tab_LeaveCHG, "yes", "yes")
func.graph.effsize(tab_LeaveCHH, "yes", "no")
func.graph.effsize(tab_LeaveCpG, "yes", "no")

dev.off()




###-----------------------------
###
###  Power analysis Suppl. Materials
###
###-----------------------------

library(wmwpow)
```

```
shiehpow(n = 6, m = 6, alpha = 0.05, dist = "norm", p = 0.675)

# effect size r = 0.2, corresponds to effect size odds = 2.0969

# conversion from r to odds

# https://www.psychometrica.de/effect_size.html



###---------------------------

###   Graph Power analysis

###   of the Mann-whitney tests

###---------------------------

r.output <- seq(0, 1, 0.1)

power.output <- numeric(11)


## r= 0.1, odd = 1.4399

power.output[1] <- wmwpowp(n = 6, m = 6, distn = "norm", alpha = 0.05, nsims = 10000, wmwodds = 1)$empirical_power

power.output[2] <- wmwpowp(n = 6, m = 6, distn = "norm", alpha = 0.05, nsims = 10000, wmwodds = 1.4399)$empirical_power

power.output[3] <- wmwpowp(n = 6, m = 6, distn = "norm", alpha = 0.05, nsims = 10000, wmwodds = 2.0969)$empirical_power

power.output[4] <- wmwpowp(n = 6, m = 6, distn = "norm", alpha = 0.05, nsims = 10000, wmwodds = 3.1294)$empirical_power

power.output[5] <- wmwpowp(n = 6, m = 6, distn = "norm", alpha = 0.05, nsims = 10000, wmwodds = 4.8706)$empirical_power

power.output[6] <- wmwpowp(n = 6, m = 6, distn = "norm", alpha = 0.05, nsims = 10000, wmwodds = 8.1205)$empirical_power

power.output[7] <- wmwpowp(n = 6, m = 6, distn = "norm", alpha = 0.05, nsims = 10000, wmwodds = 15.1909)$empirical_power

power.output[8] <- wmwpowp(n = 6, m = 6, distn = "norm", alpha = 0.05, nsims = 10000, wmwodds = 35.0143)$empirical_power

power.output[9] <- wmwpowp(n = 6, m = 6, distn = "norm", alpha = 0.05, nsims = 10000, wmwodds = 126.0651)$empirical_power

power.output[10] <- wmwpowp(n = 6, m = 6, distn = "norm", alpha = 0.05, nsims = 10000, wmwodds = 1790.1488)$empirical_power
```

```
power.output[11] <- wmwpowp(n = 6, m = 6, distn = "norm", alpha = 0.05, nsims = 10000, wmwodds = 113857319384.089)$empirical_power

plot(power.output ~ r.output, ylab = "Power", xlab = "Effect size, r")
abline(v = c(0.3, 0.5, 0.7), lty = 3, col = "gray60")

#########################################################
###
###    - 3 Correlation of Phenotypicand Methylation Data
###
#########################################################

### load Data
## From section 1 of the script
tab_effsize_Pheno <- read.table("tab_effsize_Pheno.txt")
Pheno <- read.csv("ENSFEA2 DATA working Pierick.csv", sep = ";")

## From section 2 of the script
tab_ApexCHG <- read.table("tab_ApexCHG.txt")
tab_ApexCHH <- read.table("tab_ApexCHH.txt")
tab_ApexCpG <- read.table("tab_ApexCpG.txt")

tab_LeaveCHG <- read.table("tab_LeaveCHG.txt")
tab_LeaveCHH <- read.table("tab_LeaveCHH.txt")
tab_LeaveCpG <- read.table("tab_LeaveCpG.txt")
```

```
### in Apex Methyl protocols ID corresponds to apex_tube in Pheno

### in Leaves Methyl protocols ID corresponds to leaves_tube in Pheno

##  Sort the two datasets to match IDs

Apex.CHG.sort <- Apex.CHG[order(Apex.CHG$ID), ]

Pheno.sort <- Pheno[order(Pheno$apex_tube), ]

Apex.CHG.sort <- data.frame(Apex.CHG.sort, Pheno.sort)




###

### Correlations between Phenotypic trait and Methylation ACP coords

### for cases where there is an effect of treatment on methyl ACP coords

###


###---------------------

## Apex

###---------------------


### Prepare table

traits <- c("Ramification", "Mean_Lg_node", "Diameter", "Total_leaves_number", "SLA", "Height")

Tab_apex_corr <- expand.grid(traits, c("Apex.CHG", "Apex.CHH", "Apex.CHH", "Apex.CHH",
"Apex.CpG", "Apex.CpG", "Apex.CpG"))

# Tab_apex_corr <- data.frame(Tab_apex_corr[, 2], Tab_apex_corr[, 1])

Tab_apex_corr <- data.frame(Tab_apex_corr,

                        c(rep("Ji98", 6), rep("165E", 6), rep("Ji98", 6), rep("Si50", 6),

                          rep("165E", 6), rep("Ji75", 6), rep("Si50", 6)),

                        c(rep("Dim.5", 6), rep("Dim.4", 6), rep("Dim.3", 6), rep("Dim.10", 6),
```

```
                    rep("Dim.3", 6), rep("Dim.12", 6), rep("Dim.4", 6)))

Cor.rho <- numeric(length(Tab_apex_corr[, 1]))
CiL <- numeric(length(Tab_apex_corr[, 1]))
CiU <- numeric(length(Tab_apex_corr[, 1]))
Tab_apex_corr <- data.frame(Tab_apex_corr, Cor.rho, CiL, CiU)
dimnames(Tab_apex_corr)[[2]] <- c("traits", "Meth.protocol", "Line", "Dim", "Cor.rho", "CiL", "CiU")


###
### Function that calculates correlation coefficient and CI between phenotypic traits and Methylation PCA coordinates
### for Apex data
###
func.spearm.ci.traits_Methyl <- function(DATA, LINE, DIM){


  ##  Sort datasets to match IDs
  temp.data.sort <- DATA[order(DATA$ID), ]
  Pheno.sort <- Pheno[order(Pheno$apex_tube), ]
  temp.data.sort <- data.frame(temp.data.sort, Pheno.sort)

  ##  Temp output tab
  temp.output <- matrix(nrow = 6, ncol = 3)
  ##  Calculate for each of the 6 traits
  for(i in 1 : 6){

    temp <- spearman.ci(temp.data.sort[, (ncol(temp.data.sort) - 6) + i][temp.data.sort$Line == LINE],
               temp.data.sort[, DIM][temp.data.sort$Line == LINE],
               nrep = 5000)
```

```
    temp.output[i, ] <- c(as.numeric(temp$estimate), as.numeric(temp$conf.int))

  }
  return(temp.output)
}

### Estimates correlations (CI)
ap1 <- func.spearm.ci.traits_Methyl(Apex.CHG, "Ji98", "Dim.5")
ap2 <- func.spearm.ci.traits_Methyl(Apex.CHH, "165E", "Dim.4")
ap3 <- func.spearm.ci.traits_Methyl(Apex.CHH, "Ji98", "Dim.3")
ap4 <- func.spearm.ci.traits_Methyl(Apex.CHH, "Si50", "Dim.10")
ap5 <- func.spearm.ci.traits_Methyl(Apex.CpG, "165E", "Dim.3")
ap6 <- func.spearm.ci.traits_Methyl(Apex.CpG, "Ji75", "Dim.12")
ap7 <- func.spearm.ci.traits_Methyl(Apex.CpG, "Si50", "Dim.4")

ap <- rbind(ap1, ap2, ap3, ap4, ap5, ap6, ap7)
Tab_apex_corr[, 5 : 7] <- ap

write.csv(Tab_apex_corr, file = "Tab_apex_corr.csv")

#-----------------------
## Leaves Results
#-----------------------

### Prepare table
traits <- c("Ramification", "Mean_Lg_node", "Diameter", "Total_leaves_number", "SLA", "Height")
Tab_leave_corr <- expand.grid(traits, c("Leave.CHG", "Leave.CHH", "Leave.CpG", "Leave.CpG"))
```

```
Tab_leave_corr <- data.frame(Tab_leave_corr,
                             c(rep("Ji75", 6), rep("Ji75", 6), rep("165E", 6), rep("Ji98", 6)),
                             c(rep("Dim.5", 6), rep("Dim.6", 6), rep("Dim.12", 6), rep("Dim.1", 6)))


Cor.rho <- numeric(length(Tab_leave_corr[, 1]))
CiL <- numeric(length(Tab_leave_corr[, 1]))
CiU <- numeric(length(Tab_leave_corr[, 1]))
Tab_leave_corr <- data.frame(Tab_leave_corr, Cor.rho, CiL, CiU)
dimnames(Tab_leave_corr)[[2]] <- c("traits", "Meth.protocol", "Line", "Dim", "Cor.rho", "CiL", "CiU")

###
### Function that calculates correlation coefficient and CI between phenotypic traits and Methylation PCA coordinates
### for Leaves data
###
func.spearm.ci.traits_Methyl <- function(DATA, LINE, DIM){

  ##  Sort datasets to match IDs
  temp.data.sort <- DATA[order(DATA$ID), ]
  Pheno.sort <- Pheno[Pheno$leaves_tube %in% Leaves.CHG$ID, ]
  Pheno.sort <- Pheno.sort[order(Pheno.sort$leaves_tube), ]
  temp.data.sort <- data.frame(temp.data.sort, Pheno.sort)

  ##  Temp output tab
  temp.output <- matrix(nrow = 6, ncol = 3)
  ##  Calculate for each of the 6 traits
  for(i in 1 : 6){

    temp <- spearman.ci(temp.data.sort[, (ncol(temp.data.sort) - 6) + i][temp.data.sort$Line == LINE],
               temp.data.sort[, DIM][temp.data.sort$Line == LINE],
```

```
                     nrep = 5000)


    temp.output[i, ] <- c(as.numeric(temp$estimate), as.numeric(temp$conf.int))


  }
  return(temp.output)
}


### Estimates correlations (CI)
le1 <- func.spearm.ci.traits_Methyl(Leaves.CHG, "Ji75", "Dim.5")
le2 <- func.spearm.ci.traits_Methyl(Leaves.CHH, "Ji75", "Dim.6")
le3 <- func.spearm.ci.traits_Methyl(Leaves.CpG, "165E", "Dim.12")
le4 <- func.spearm.ci.traits_Methyl(Leaves.CpG, "Ji98", "Dim.1")


le <- rbind(le1, le2, le3, le4)
Tab_leave_corr[, 5 : 7] <- le


write.csv(Tab_leave_corr, file = "Tab_leave_corr.csv")




###
### Output tables
###


##  Load dataset
Tab_apex_corr <- read.csv2(file = "Tab_apex_corr.csv", dec = ".")
Tab_leave_corr <- read.csv2(file = "Tab_leave_corr.csv", dec = ".", sep = ",")


levels(Tab_apex_corr$traits) <- c("Number of branches", "Mean internode length", "Diameter", "Total leaves number", "SLA", "Height")
```

```
levels(Tab_leave_corr$traits) <- c("Number of branches", "Mean internode length", "Diameter", "Total leaves number", "SLA", "Height")



### Apex

Tab_apex_corr_sorted <- Tab_apex_corr[order(Tab_apex_corr$traits), ]

Tab_apex_corr_sorted[, (ncol(Tab_apex_corr_sorted) - 2) : ncol(Tab_apex_corr_sorted)] <- round(Tab_apex_corr_sorted[, (ncol(Tab_apex_corr_sorted) - 2) : ncol(Tab_apex_corr_sorted)], 3)

Tab_apex_corr_sorted[, "95% CI"] <- paste(Tab_apex_corr_sorted$CiL, Tab_apex_corr_sorted$CiU, sep = "; ")

Tab_apex_corr_sorted <- Tab_apex_corr_sorted[, -c(1,7,8)]

write.csv(x = Tab_apex_corr_sorted, file = "Tab_apex_corr_sorted.csv")


### Leaves

Tab_leave_corr_sorted <- Tab_leave_corr[order(Tab_leave_corr$traits), ]

Tab_leave_corr_sorted[, (ncol(Tab_leave_corr_sorted) - 2) : ncol(Tab_leave_corr_sorted)] <- round(Tab_leave_corr_sorted[, (ncol(Tab_leave_corr_sorted) - 2) : ncol(Tab_leave_corr_sorted)], 3)

Tab_leave_corr_sorted[, "95% CI"] <- paste(Tab_leave_corr_sorted$CiL, Tab_leave_corr_sorted$CiU, sep = "; ")

Tab_leave_corr_sorted <- Tab_leave_corr_sorted[, -c(1,7,8)]

write.csv2(x = Tab_leave_corr_sorted, file = "Tab_leave_corr_sorted.csv")



### -------------------

###

###   FIGURE 4

###

### -------------------


### Check if 0 included in CI (1 = zero included in CI)

### Apex data

vect.ci.incl.Tab.Apex <- numeric(length(Tab_apex_corr[, 1]))
```

```
for(i in 1 : length(vect.ci.incl.Tab.Apex)){
  # if coef > 0, 0 not included if conf.low > 0
  # 1 means 0 included
  if(isTRUE(Tab_apex_corr$Cor.rho[i] > 0) == T){

    vect.ci.incl.Tab.Apex[i] <- ifelse(test = Tab_apex_corr$CiL[i] > 0, 0, 1)

  } else if(isTRUE(Tab_apex_corr$Cor.rho[i] < 0) == T){    # if effsize < 0, 0 not included if conf.high < 0

    vect.ci.incl.Tab.Apex[i] <- ifelse(test = Tab_apex_corr$CiU[i] < 0, 0, 1)

  } else if(isTRUE(Tab_apex_corr$Cor.rho[i] == 0) == T){

    vect.ci.incl.Tab.Apex[i] <- 1

  }
}

### Leaves data
vect.ci.incl.Tab.Leave <- numeric(length(Tab_leave_corr[, 1]))
for(i in 1 : length(vect.ci.incl.Tab.Leave)){
  # if coef > 0, 0 not included if conf.low > 0
  # 1 means 0 included
  if(isTRUE(Tab_leave_corr$Cor.rho[i] > 0) == T){

    vect.ci.incl.Tab.Leave[i] <- ifelse(test = Tab_leave_corr$CiL[i] > 0, 0, 1)

  } else if(isTRUE(Tab_leave_corr$Cor.rho[i] < 0) == T){    # if effsize < 0, 0 not included if conf.high < 0

    vect.ci.incl.Tab.Leave[i] <- ifelse(test = Tab_leave_corr$CiU[i] < 0, 0, 1)
```

```
  } else if(isTRUE(Tab_leave_corr$Cor.rho[i] == 0) == T){

    vect.ci.incl.Tab.Leave[i] <- 1

  }
}


###
### Plot FIGURE 4
###

png(filename = "Plot_Corr_Pheno_Meth_Bytraits.png", width = 24, height = 14, units = 'in', res = 300)
{

  par(mfrow = c(1, 2), mar = c(5, 8, 3, 1), xpd = F, cex = 1.5)
  ### Plot Apex

  ## y.axis position
  y.axis.pos <- 1 : nrow(Tab_apex_corr)


  plot(y.axis.pos ~ Tab_apex_corr$Cor.rho[order(Tab_apex_corr$traits)], xlim = c(- 0.95, 1.3),
       ylab = "", xlab = "Spearman correlation coefficient [95% CI]", type = "n", yaxt = "n",
       cex.lab = 2, cex.axis = 1.5)

  abline(v = 0, lwd = 2)
  abline(v = c(-0.3, -0.5, -0.7, 0.3, 0.5, 0.7), lty = 3, col = "gray50")

  polygon(y = c(7.5, 7.5, 14.5, 14.5), x = c(-1, 1.36, 1.36, -1),  col = alpha("gray", 0.2), border = NA)
  polygon(y = c(21.5, 21.5, 28.5, 28.5), x = c(-1, 1.36, 1.36, -1),  col = alpha("gray", 0.2), border = NA)
```

```
polygon(y = c(35.5, 35.5, 42.5, 42.5), x = c(-1, 1.36, 1.36, -1),  col = alpha("gray", 0.2), border = NA)

segments(y0 = y.axis.pos, x0 = Tab_apex_corr$CiL[order(Tab_apex_corr$traits)],
     x1 = Tab_apex_corr$CiU[order(Tab_apex_corr$traits)], lwd = 2)

points(y.axis.pos ~ Tab_apex_corr$Cor.rho[order(Tab_apex_corr$traits)],
    pch = ifelse(vect.ci.incl.Tab.Apex[order(Tab_apex_corr$traits)] == 0, 19, 1),
    cex = ifelse(vect.ci.incl.Tab.Apex[order(Tab_apex_corr$traits)] == 0, 1.3, 1))

mtext(text = c("Number\nof\nbranches", "Mean\ninternode\nlength", "Diameter",
"Total\nleaves\nnumber", "SLA", "Height"),
   side = 2, at = c(4, 11, 18, 25, 32, 39), cex = 2.3, line = 3, las = 2, adj = 0.5)

mtext(text = "Traits", side = 2, line = 6, cex = 2.5)

mtext(text = "A", side = 3, line = 1,
   adj = 1, font = 2, cex = 2.5)

mtext(text = "Apex", side = 3, line = 1, cex = 2.5, font = 2)

text(labels = c("CHG Ji98 Dim.5", "CHH 165E Dim.4", "CHH Ji98 Dim.3", "CHH Si50 Dim.10",
        "CpG 165E Dim.3", "CpG Ji75 Dim.12", "CpG Si50 Dim.4"),
  y = y.axis.pos, x = 1, cex = 0.7, adj = 0)



### Plot Leaves

## y.axis position
y.axis.pos <- 1 : nrow(Tab_leave_corr)

plot(y.axis.pos ~ Tab_leave_corr$Cor.rho[order(Tab_leave_corr$traits)], xlim = c(- 0.95, 1.3),
```

```
      ylab = "", xlab = "Spearman correlation coefficient [95% CI]", type = "n", yaxt = "n",
      cex.lab = 2, cex.axis = 1.5, cex = 2)

  abline(v = 0, lwd = 2)
  abline(v = c(-0.3, -0.5, -0.7, 0.3, 0.5, 0.7), lty = 3, col = "gray50")

  polygon(y = c(4.5, 4.5, 8.5, 8.5), x = c(-1, 1.37, 1.37, -1),  col = alpha("gray", 0.2), border = NA)
  polygon(y = c(12.5, 12.5, 16.5, 16.5), x = c(-1, 1.37, 1.37, -1),  col = alpha("gray", 0.2), border = NA)
  polygon(y = c(20.5, 20.5, 24.5, 24.5), x = c(-1, 1.37, 1.37, -1),  col = alpha("gray", 0.2), border = NA)

  segments(y0 = 1 : length(Tab_leave_corr[, 1]),
        x0 = Tab_leave_corr$CiL[order(Tab_leave_corr$traits)], x1 =
Tab_leave_corr$CiU[order(Tab_leave_corr$traits)],
        lwd = 2)
  points(y.axis.pos ~ Tab_leave_corr$Cor.rho[order(Tab_leave_corr$traits)],
       pch = ifelse(vect.ci.incl.Tab.Leave[order(Tab_leave_corr$traits)] == 0, 19, 1),
       cex = ifelse(vect.ci.incl.Tab.Leave[order(Tab_leave_corr$traits)] == 0, 1.3, 1))

  mtext(text = "B", side = 3, line = 1,
      adj = 1, font = 2, cex = 2.5)
  mtext(text = "Leaves", side = 3, line = 1, cex = 2.5, font = 2)

  mtext(text = c("Number\nof\nbranches", "Mean\ninternode\nlength", "Diameter",
"Total\nleaves\nnumber", "SLA", "Height"),
      side = 2, at = c(2.5, 6.5, 10.5, 14.5, 18.5, 22.5), cex = 2.3, line = 3, las = 2, adj = 0.5)

  text(labels = c("CHG Ji75 Dim.5", "CHH Ji75 Dim.6", "CpG 165E Dim.12", "CpG ji98 Dim.1"),
      y = y.axis.pos, x = 1, cex = 0.7, adj = 0)


}
dev.off()
```

```
### --------------------------------
###
### Power analysis spearman corr
###
### --------------------------------
library(pwr)
##  here we use the spearman correlation coefficient (rs) as if it were pearson coefficient (rp).
##  Myers, L., & Sirois, M. J. (2006). Spearman Correlation Coefficients, Differences between.
Encyclopedia of Statistical Sciences. doi:10.1002/0471667196.ess5050.pub2


r.test <- seq(0, 1, 0.05)
p.output <- pwr.r.test(n = 12, r = r.test, sig.level = 0.05)$power


tiff(filename = "Plot_power_Correlation.tiff", width = 12, height = 12, units = 'in', res = 300)


par(mar = c(5,4,2,2))
plot(p.output ~ r.test, xlab = "Correlation coefficient (rs)", ylab = "Power")
abline(v = c(0.3, 0.5, 0.7), lty = 3, col = "gray60")


dev.off()




## END
```
